# Identifying Behaviour Change Techniques in Cancer Nutrition Interventions and Their Implementation Contexts: A Systematic Review

**DOI:** 10.3390/nu18020242

**Published:** 2026-01-12

**Authors:** Shuang Liang, Niamh C. Fanning, Amanda Landers, Helen Brown, Catriona Rother, Fong Fu, Guillaume Fontaine, April Morrow, Natalie Taylor

**Affiliations:** 1 Implementation to Impact, School of Population Health, Faculty of Medicine and Health, The University of New South Wales, Sydney, NSW 2052, Australia; april.morrow@unsw.edu.au (A.M.); natalie.taylor@unsw.edu.au (N.T.); 2 Department of Medicine, University of Otago, Christchurch 8011, New Zealand; fanningn@tcd.ie (N.C.F.); amanda.landers@otago.ac.nz (A.L.); fufo2743@student.otago.ac.nz (F.F.); 3 Nurse Maude Hospice Palliative Care Service, Nurse Maude Association, Christchurch 8014, New Zealand; helen.brown@nursemaude.org.nz; 4 Wellington Blood and Cancer Centre, Te Whatu Ora Capital, Coast and Hutt Valley, Wellington 6021, New Zealand; catriona.rother@hotmail.com; 5 Methodological and Implementation Research, Ottawa Hospital Research Institute, Ottawa, ON K1Y 4E9, Canada; guil.fontaine@mcgill.ca; 6 Ingram School of Nursing, Faculty of Medicine and Health Sciences, McGill University, Montreal, QC H3G 2M1, Canada; 7 Centre for Clinical Epidemiology, Lady Davis Institute for Medical Research, Sir Mortimer B. Davis Jewish General Hospital, Montreal, QC H3T 1E1, Canada; 8 Kirby Institute, University of New South Wales, Sydney, NSW 2052, Australia

**Keywords:** health systems, implementation science, mixed-methods, nutrition care, theoretical domains framework

## Abstract

Background/Objectives: Effective nutritional care is a critical component of oncology treatment. Behaviour change techniques (BCTs) are key elements that drive individual behaviour change and are commonly identified within clinical interventions and implementation strategies. Despite their relevance, the application of BCTs in oncology nutrition has not been systematically examined. This systematic review aimed to identify and synthesise the use of BCTs in nutrition interventions and their implementation strategies within adult oncology care. Methods: A search across 10 electronic databases was conducted from inception up to December 2024. Data extraction focused on intervention characteristics, plus implementation barriers, strategies, and outcomes, which were coded using relevant established taxonomies, i.e., Theoretical Domains Framework, Behaviour Change Techniques, and Proctor’s outcomes framework. Study quality was assessed using the QuADS tool. Results: After screening 4055 abstracts and 165 full texts, 38 articles representing 31 unique studies were included. Nutrition interventions (*n* = 31) spanned across risk screening/assessment, care planning/pathways, nutritional monitoring, and support guidelines. Among the 19 interventions that incorporated BCTs targeting patients and/or healthcare professionals (HCPs), 20 unique BCTs were identified across 97 instances. Separately, implementation strategies reported in 25 of the 38 articles included 96 BCT instances (18 unique) targeting HCPs. BCTs were frequently observed alongside positive outcomes across patient, service, and implementation levels. Conclusions: Application of BCTs in oncology nutrition practice remains implicit and inconsistent. For clinical practice, more explicit specifications of BCTs may support better integration of nutrition care in routine oncology settings. Future research is warranted to test the effectiveness of specific BCTs and their combinations. This can be supported by standardised reporting of intervention content and implementation strategies which can enable identification of patterns of success and optimise replicability.

## 1. Introduction

Cancer remains a leading cause of mortality worldwide, with over 35 million new cases projected in 2050, representing a 77% increase from the close to 20 million cases in 2022 [1]. While high-income countries are expected to experience the greatest absolute increase in cancer incidence, the most dramatic proportional rises are anticipated in low- and middle-income countries, where healthcare resources are often most limited [2]. This growing burden underscores the urgent need for scalable and cost-effective strategies to improve cancer care outcomes globally [3].

Malnutrition is highly prevalent among oncology patients and is associated with poorer treatment outcomes, reduced quality of life, and increased mortality [4]. In response, nutrition interventions, such as risk assessment and screening, care plans and pathways, nutritional monitoring, and adherence to evidence-based guidelines, are increasingly recognised as essential components of comprehensive cancer care, yet their integration into routine oncology practice remains limited [5].

Despite growing emphasis on nutritional care interventions, their implementation is often hindered by persistent barriers such as the absence of standardised guidelines, time constraints, and limited awareness among clinicians and patients [6]. Implementation science offers a structured approach to understanding and addressing these barriers, thereby supporting the successful integration of evidence-based practices [7]. Within this field, the Theoretical Domains Framework (TDF) [8] provides a systematic method for identifying and categorising implementation barriers [9]. Once these barriers are understood, targeted strategies can be developed to facilitate behaviour change among key actors, thereby enhancing the adoption of innovations and the effective translation of evidence into practice.

Behaviour change techniques (BCTs) are the active components of interventions designed to influence behaviour at the individual level [10]. They are used to address specific barriers faced by a given population (e.g., HCPs or patients). BCTs serve as building blocks for designing strategies that support both HCPs and patients in vital behaviour changes necessary for the successful uptake of novel interventions [11,12]. Despite the relevance of BCTs in the design and implementation of nutrition interventions, there is a lack of synthesis on how they are applied in oncology settings. Specifically, there is limited understanding of how BCTs are operationalised, which techniques are most effective, and whether they address known implementation barriers. Since these barriers can be coded using the TDF, we leveraged the TDF-BCT Theory and Techniques Tool to explore whether the observed sequence of change aligned with established behavioural mechanism.

This systematic review aimed to systematically identify, code, and synthesise evidence on the use of BCTs in nutrition interventions and their implementation as delivered in adult oncology care, using established theoretical frameworks to support analytical synthesis. The specific objectives were to:

1. Identify barriers faced by HCPs in implementing nutrition interventions, as reported in the literature, and code these barriers to the TDF;
2. Identify the BCTs used in nutrition interventions and their implementation strategies;
3. Identify associated outcomes at the patient, service, and implementation levels;
4. Explore how TDF-BCT linkages align with established mechanisms of behaviour change.

## 2. Materials and Methods

### 2.1. Design and Registration

This systematic review was conducted in accordance with the Preferred Reporting Items for Systematic Reviews and Meta-Analyses (PRISMA) guidelines [13]. The review protocol was registered prospectively in the PROSPERO database (CRD42023454210) [14]. A PRISMA checklist [13] was completed (Supplementary File S1).

### 2.2. Searches

A comprehensive search [15] was conducted across 10 electronic databases up to week 4, December 2024. These included: MEDLINE, AMED, APA PsychINFO, CINAHL, Embase, Emcare, ERIC, Joanna Briggs Institute (JBI), Ovid Nursing Database, Global Health and EBM Reviews: Cochrane Central Register of Controlled Trials. Search terms were developed in collaboration with a professional librarian to ensure comprehensive coverage of key concepts, including nutrition, cancer, and implementation.

### 2.3. Study Inclusion and Exclusion Criteria

Inclusion criteria included:

- Population: adults diagnosed with any type of cancer;
- Intervention: the implementation of nutrition interventions in routine clinical practice;
- Comparator: with or without a comparator group;
- Outcomes: implementation, service, and/or clinical outcomes;
- Study design/source: Range of methodologies including randomised control trials, quasi-experimental, retrospective controlled, observational, mixed-methods, qualitative, and implementation studies were included to enable a more comprehensive synthesis of evidence related to the implementation of nutrition interventions in cancer care.

Exclusion criteria included:

- Population: non-adult populations, cancer survivors, or individuals without cancer;
- Outcomes: lacking implementation components or relevant outcomes;
- Study design/source: Non-original research or studies without accessible full-texts, and editorials, reviews, letters and anecdotal reports, non-English language publications.

When protocol papers were identified, additional manual searches were conducted to locate subsequent publications reporting study outcomes. Reference lists of relevant review articles were also screened to identify further eligible studies.

The review process was managed using Covidence. Titles, abstracts, and full texts were screened independently by 2 reviewers (NCF, AL, FF, CR, HB), with disagreements resolved through discussion and/or consultation with a third reviewer. Inter-rater reliability was monitored throughout the screening process by comparing reviewer decisions and resolving discrepancies to ensure consistent application of eligibility criteria. For quality control, a random sample of 15 full text articles was independently reviewed by implementation science experts (AM and SL), with 100% agreement observed.

### 2.4. Study Quality Assessment

Study quality was assessed using the Quality Assessment with Diverse Studies (QuADS) tool [16]. Two independent reviewers scored each study (maximum score: 39). If scores differed by ≤5 points, an average was calculated; discrepancies >5 points were resolved through discussion.

### 2.5. Data Extraction Strategy

Following a similar approach to Morrow et al. [17], this review focused on identifying BCTs embedded within cancer-related nutrition interventions and within implementation strategies (i.e., strategies used to support the adoption of the nutrition interventions). Extracted data included study characteristics (design, sample size, setting, participants), intervention details, implementation barriers, strategies, and outcomes at the implementation, service, and patient levels. Generic data extraction was conducted by 1 reviewer and independently verified by a second. Extraction related to implementation science were performed by researchers with expertise in the field (SL, AM), while clinician researchers were responsible for extracting data related to clinical outcomes (NCF, AL, FF, CR, HB). Coding to the TDF, BCT Taxonomy v1 [18], and Proctor’s outcomes framework was conducted independently by reviewers (SL, AM). Discrepancies were resolved through discussion and, where necessary, consultation with a third reviewer (NT). To further ensure coding reliability, NT independently reviewed a random 10% sample of the coded data. The data extraction and coding template was developed and pilot tested using REDCap (Research Electronic Data Capture) [19] and Microsoft Excel [20] prior to application (SL, NCF).

### 2.6. Data Synthesis and Presentation

Data was synthesised using frequency counts and descriptive statistics. Where applicable, the TDF was used to categorise implementation barriers, with relevant domains and constructs selected based on study context [9]. BCTs embedded in interventions targeting patients and/or HCPs, and implementation strategies targeting HCPs, were identified and coded using established taxonomies [21]. A Sankey diagram was generated using SankeyMATIC [22] to visualise the spectrum of BCTs. Outcomes were categorised using Proctor’s framework into implementation, service, and patient-level outcomes [23], and were further classified as positive, neutral or negative based on author interpretation. BCTs linked to positive outcomes were examined in relation to their target populations, i.e., patients and/or HCPs.

To explore mechanistic pathways, full sequences of observed change were analysed in studies where BCTs were present in both intervention and implementation strategies and outcomes were reported at both implementation/service and patient levels. In addition, the TDF-BCT linkage tool [24] was used to map each BCT to corresponding TDF constructs, assessing whether observed linkages aligned with existing evidence or suggested novel associations.

## 3. Results

### 3.1. Study Characteristics

**Figure 1:**
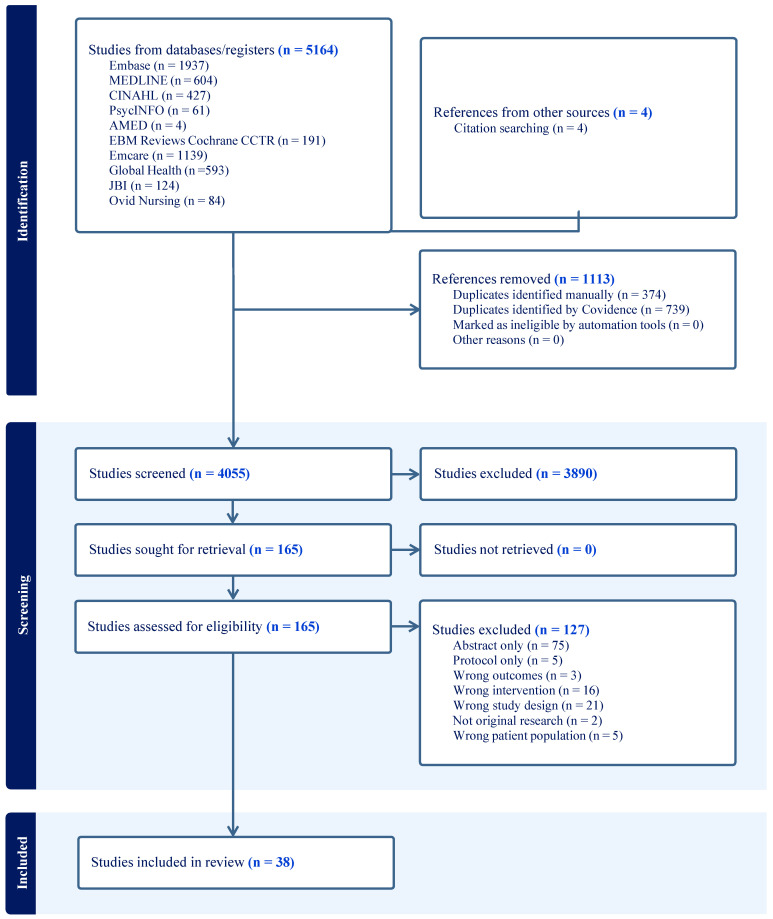
PRISMA flow diagram for study selection.

Table 1 provides an overview of the study characteristics. Of the 5168 records identified through the systematic search, 4055 abstracts met the inclusion criteria, and 165 records were screened at the full text level. A total of 38 full texts, derived from 31 unique studies, were included in the final review. The study selection process is presented in the PRISMA flow diagram (Figure 1). Across the 31 unique studies, the nutrition interventions implemented were categorised into 4 main types: risk screening/assessment (*n* = 5), care planning/pathways (*n* = 17), nutritional monitoring (*n* = 3), and support guidelines (*n* = 6).

Most articles reported on studies conducted in Australia (*n* = 14), followed by the United States (*n* = 6), China (*n* = 5), France (*n* = 3), Singapore (*n* = 2), and the United Kingdom (*n* = 2). One article reported on separate studies conducted in Brazil, Croatia, Italy, Malaysia, Spain, and the Netherlands. Most articles focused on patient populations (*n* = 28). Nine articles addressed both HCP and patient perspectives [25,26,27,28,29,30,31,32,33], while 1 article specifically targeted oncologists [34]. The most common research design was pre-post (retrospective and prospective; *n* = 18), followed by RCTs (*n* = 7), including stepped-wedge and cluster-randomised designs. Other study designs included cross sectional, observational, and process evaluation studies. The risk of bias scores ranged from 25.5 (65%) to 37.5 (96%), with a median score of 31.5 (81%).

### 3.2. Barriers to Implementation (TDF)

Twenty-three articles (61%) explicitly reported barriers to the provision of nutritional care in oncology settings. Across these articles, 27 unique barriers were identified in a total of 62 instances and mapped to 11/14 domains of the TDF. A detailed breakdown is provided in Table 2.

The most frequently identified domain was ‘Environmental Context and Resources’, reported 39 times across 23 (61%) articles. This domain encompassed a range of systemic and logistical challenges that hinder effective nutritional care. Key barriers within this domain included a lack of standardised nutritional care guidelines and protocols, time constraints for clinicians, and inadequate resourcing.

The second most identified domain was ‘Knowledge,’ identified 9 times across 8 (21%) articles. Barriers within this domain highlighted critical gaps in both clinician and patient knowledge regarding nutritional care. Clinicians often lacked awareness of current nutritional care guidelines and procedures, whilst patients and their caregivers were uninformed about the importance of nutrition in cancer treatment.

Less frequently identified barriers included those within the ‘Skills’ domain, which highlighted a gap in clinicians’ ability to effectively assess and manage nutritional care, potentially compromising the quality of patient care. The ‘Social Influences’ domain revealed communication challenges among healthcare staff that could hinder the coordination required for delivering comprehensive nutritional care. Additionally, barriers related to ‘Beliefs About Capabilities’ suggest that clinicians might lack confidence in supporting patients to modify dietary behaviours, whilst patients themselves may feel uncertain about their ability to make such changes. Barriers related to ‘Beliefs About Consequences’ suggest that either group may not fully recognise the importance of nutrition as a vital component of cancer treatment.

**Table 1:** Study characteristics and nutrition interventions.

Author, Year, Country	Design, Setting	Participants, Sample Size	Nutrition Intervention	Risk of Bias Score
Adriaans 2022 [35] Netherlands	Prospective observational Major hospital (*n* = 1)	*n* = 84 patients with potentially curable oesophageal cancer planned for surgery	*Nutritional monitoring* Nutritional monitoring with follow-up from diagnosis to surgery	32.5
Atkins 2019 [36] Australia	Implementation study Tertiary cancer centre	*n* = 20 patients with haematological malignancies receiving autologous haemopoetic stem cell transplant	*Nutritional care planning/pathways* Nutrition care pathway provided by dietitians	32
Beck 2020 [26] Australia	Stepped-wedge randomised control trial Major hospitals (*n* = 5)	*n* = 303 patients with head and neck cancer *n* = 24 radiotherapy dietitians	*Nutritional care planning/pathways* Dietitian-delivered health behavioural counselling intervention aimed at reducing malnutrition based on motivational and behavioural change principles ‘Eating As Treatment (EAT)’	37
Beck 2021 [25] Australia	Process evaluation Major hospitals (*n* = 5)	*n* = 107 patients with head and neck cancer *n* = 20 dietitians	*Nutritional care planning/pathways* Dietitian-delivered health behavioural counselling intervention aimed at reducing malnutrition based on motivational and behavioural change principles ‘Eating As Treatment (EAT)’	34.5
Belluomini 2024 [37] Italy	Prospective observational trial University hospital oncology unit (*n* = 1)	*n* = 94 patients with a diagnosis of thoracic malignancies at any stage	*Nutritional risk screening/assessment* Oncologists-delivered early nutritional screening and referral	24
Blake 2022 [38] Australia	Retrospective pre-post implementation study Tertiary/quaternary cancer care centre	*n* = 64 pre- vs. *n* = 47 post- patients with head and neck squamous cell carcinoma classed as being at nutritional risk	*Nutritional care planning/pathways* Pre-treatment model of nutritional care consisting of dietary counselling and commencement of proactive supplementary enteral nutrition in addition to normal oral intake	30
Britton 2019 [39] Australia	Stepped-wedge cluster randomised controlled trial Major hospitals (*n* = 5)	*n* = 307 (*n* = 156 intervention vs. *n* = 151 control) patients with head and neck cancer	*Nutritional care planning/pathways* Dietitian-delivered health behavioural counselling intervention aimed at reducing malnutrition based on motivational and behavioural change principles ‘Eating As Treatment (EAT)’	35
Britton 2024 [40] Australia	Exploratory analysis of stepped-wedge cluster randomised controlled trial Major hospitals (*n* = 5)	*n* = 307 (*n* = 156 intervention vs. *n* = 151 control) patients with head and neck cancer	*Nutritional care planning/pathways* Dietitian-delivered health behavioural counselling intervention aimed at reducing malnutrition based on motivational and behavioural change principles ‘Eating As Treatment (EAT)’	32.5
Carr 2022 [41] USA	Retrospective pre-post study Surgical service	*n* = 404 (*n* = 217 pre- vs. *n* = 187 post-) patients with oesophageal cancer	*Nutritional care planning/pathways* Perioperative nutrition programme	27
Chen 2012 [42] Singapore	Pre-post study Major hospital (acute care) (*n* = 1)	*n* = 24 patients with cancer (oncological and haematological malignancies)	*Nutritional risk screening/assessment* Nutritional screening practice of registered nurses using 3-MinNS nutritional screening tool	31
Cook 2023 [43] UK	Pre-post study Major hospital (*n* = 1)	*n* = 33 (*n* = 15 pre- vs. *n* = 18 post-) patients with head and neck cancer who underwent radiologically inserted gastrostomy (RIG) replacement	*Nutritional risk screening/assessment* Dietetic-led pre-RIG insertion risk assessment on nutritional outcomes and complications	34.5
Deftereos 2022 [27] Australia	Qualitative Major hospital (*n* = 4)	*n* = 23 patients from intervention group of Deftereos 2023 [44] *n* = 12 dietitians *n* = 14 multidisciplinary team members	*Nutritional care planning/pathways* Perioperative nutrition care pathway	36.5
Deftereos 2023 [44] Australia	Pre-post study Major tertiary cancer surgery centres (*n* = 3) and preoperative care centre (*n* = 1)	*n* = 35 pre- vs. *n* = 35 post- patients with upper gastrointestinal (UGI) cancer planned for curative intent surgery	*Nutritional care planning/pathways* Perioperative nutritional care pathway	36
Den 2021 [45] Australia	Retrospective pre-post study Tertiary cancer centre (*n* = 1)	*n* = 80 (*n* = 40 pre- vs. *n* = 40 post-) patients with surgical lower gastrointestinal and pelvic cancer	*Nutritional care planning/pathways* Evidence-based nutrition care pathway	30
Ding 2023 [46] China	Non-randomised clinical trial Hospital surgical service	*n* = 70 (*n* = 34 routine vs. *n* = 36 personalised) patients requiring oral cancer surgery for head and neck cancer	*Nutritional care planning/pathways* Routine enteral nutrition vs. personalised enteral nutrition	29
Ettori 2019 [47] France	Pre-post cohort study Tertiary care reference centre	*n* = 274 (*n* = 147 pre- vs. *n* = 128 post-) clinically ill haematology patients	*Nutritional monitoring* Computer assisted decision support system to reach predicted daily calories and protein targets	32
Findlay 2020 [28] Australia	Mixed-methods pre-post study Tertiary referral head and neck cancer unit	*n* = 98 pre- vs. *n* = 34 post- adult head and neck cancer patients undergoing radiotherapy with or without other treatment of curative intent *n* = 12 multidisciplinary members (allied health, medical, nursing)	*Nutritional monitoring* Weekly supportive care-led pre-treatment clinic and a nutrition care dashboard highlighting nutrition outcome data integrated into MDT meetings	37
Garcia-Luna 2023 [48] Spain	Prospective pre-post study Hospitals (*n* = 3)	*n* = 30 clinical histories per month over a 6-month period in head and neck, esophagogastric, biliopancreatic, and colorectal cancer	*Nutritional care planning/pathways* Integrated nutritional oncology care plan targeting malnutrition management, including MUST nutritional screening, a multidisciplinary nutrition support team (NST), consultation and online resources.	35
Gilbert 2021 [49] France	Open-label prospective stepped-wedge cluster-randomised trial Surgical hospitals (*n* = 5)	*n* = 147 patients aged 70 years or older with scheduled abdominal surgery for colorectal cancer (excluding patients hospitalised for emergency surgical resection)	*Nutritional support guidelines* Perioperative nutritional management guidelines (European Society for Clinical Nutritional & Metabolism; ESPEN)	31.5
Han 2018 [50] Malaysia	Cross-sectional Tertiary cancer care centre	*n* = 349 (initial audit); *n* = 390 (re-audit) general oncology outpatients	*Nutritional risk screening/assessment* MST nutrition screening for malnutrition; Dietitian referral if MST malnutrition score ≥ 2	25.5
Kiss 2019 [51] Australia	Prospective pre-post study MDT outpatient clinic	*n* = 91 head and neck cancer patients receiving treatment with curative intent	*Nutritional care planning/pathways* Introduction of a nutrition assistant and an 8-week training module. Partial dietitian reviews replacement with nutrition assistant.	31
Krznaric 2019 [34] Croatia	Cross-sectional Secondary or tertiary oncology setting	*n* = 128 oncologists who are members of the Croatian National Oncology Society	*Nutritional support guidelines* Nutritional guidelines for nutritional support and treatment of oncology patients, including recommendations of eicosapentaenoic acid (EPA) and megesterol acetate (MA) for 8 weeks to improve nutritional status in cancer cachexia	26.5
Ladna 2025 [52] USA	Retrospective cohort	*n* = 2143 patients underwent pancreatic resection, included individuals with chronic pancreatitis and pancreatic cancer	*Nutritional care planning/pathways* Management of exocrine pancreatic insufficiency (EPI), supported by an EPIC-based best practice alert (BPA) and smart set to optimise care, including appropriate prescription of pancreatic enzyme replacement therapy (PERT) at the correct dose	29.5
Levonyak 2021 [53] USA	Retrospective pre-post study Hospital outpatient service (*n* = 1)	*n* = 63 new patients with gastrointestinal cancer who were seen by a registered dietitian	*Nutritional care planning/pathways* Dietitian services for patients with gastrointestinal cancer	30.5
Levonyak 2022 [54] USA	Retrospective pre-post study Hospital outpatient service (*n* = 1)	*n* = 63 new patients with gastrointestinal cancer who were seen by a registered dietitian	*Nutritional care planning/pathways* Dietitian services for patients with gastrointestinal cancer	As above
Martin-McGill 2020 [55] UK	Prospective randomised pilot study with embedded qualitative component Adult neuroscience centre	*n* = 12 patient (≥16 yrs), with ECOG performance status score 0–2, histologic diagnosis of GBM and were planned to undergo radiotherapy and temozolomide chemotherapy	*Nutritional care planning/pathways* 3-month dietary intervention of medium chain triglyceride ketogenic diet or modified ketogenic diet, with the option to extend to a total of 12 months	31.5
McCarter 2018 [29] Australia	Stepped-wedge randomised controlled trial Radiotherapy departments within major metropolitan hospitals (*n* = 6)	*n* = 307 (*n* = 156 intervention vs. *n* = 151 usual care) head and neck cancer patients *n* = 8 dietitians	*Nutritional care planning/pathways* Dietitian-delivered health behavioural counselling intervention aimed at reducing malnutrition based on motivational and behavioural change principles ‘Eating As Treatment (EAT)’	37.5
Moore 2021 [56] USA	Case–control quality improvement Tertiary referral centre	*n* = 25 (prospective) matched with *n* = 25 (retrospective) patients undergoing major surgery for head and neck cancer	*Nutritional support guidelines* Nutritional protocol focuses on patient consumption of nutritional supplements perioperatively, monitored by outpatient dietitian. Early post-operative enteral nutrition with monitoring of nutritional laboratory values ‘Enhanced Recovery After Surgery (ERAS)’	36
Murray 2019 [30] Australia	Stepped-wedge cluster randomised controlled trial Radiotherapy department of major hospitals (*n* = 5)	*n* = 307 (*n* = 155 intervention vs. *n* = 151 control) head and neck cancer patients undergoing radiotherapy *n* = 29 radiotherapy dietitians	*Nutritional care planning/pathways* Dietitian-delivered health behavioural counselling intervention aimed at reducing malnutrition based on motivational and behavioural change principles ‘Eating As Treatment (EAT)’	33
Naseer 2017 [31] Singapore	Pre-post study Oncology ward in acute tertiary hospital	*n* = 24 haematology-oncology patients *n* = 26 oncology ward nurses	*Nutritional support guidelines* Best practice related to protect patients’ mealtimes	34
Pasmann 2024 [57] USA	Quality improvement Community oncology practice (*n* = 1)	*n* = 101 patients receiving oncology care in the community	*Nutritional risk screening/assessment* Nurse-led nutritional screening process using the Malnutrition Screening Tool (MST)	31
Paynter 2017 [58] Australia	Retrospective pre-post study Tertiary hospital (*n* = 1)	*n* = 36 pre- vs. *n* = 38 post- patients undergoing surgery for upper gastrointestinal (UGI) cancer	*Nutritional support guidelines* Pre-operative immune-nutrition supplement protocol	31.5
Poveda 2018 [32] Brazil	Pre-post study Cancer teaching hospital	*n* = 9 (*n* = 2 patients; *n* = 7 caregivers) in need of home-based enteral feeding *n* = 9 medical charts per audit	*Nutritional care planning/pathways* Naso-enteric feeding discharge planning	31
Senesse 2017 [59] France	Observational study Cancer centre	*n* = 3078 medical oncology inpatients hospitalised > 48 h (except those undergoing brachytherapy or expected to decease < 4 weeks)	*Nutritional support guidelines* Cancer Nutrition Programme by institution-wide multidisciplinary supportive care team to screen and manage cachexia in inpatients and outpatients in accordance with the guidelines	28
Wang 2014 [60] China	Pre-post study Medical Oncology Ward at university-affiliated Cancer Centre (*n* = 1)	*n* = 60 (*n* = 30 pre- vs. *n* = 30 post-) gastrointestinal cancer patients receiving chemotherapy	*Nutritional risk screening/assessment* Nutritional Risk Screening tool (NRS-2002) developed by the Danish Society of Parenteral and Enteral Nutrition	27
Zeng 2023 [61] China	Randomised Controlled Trial University hospital radiotherapy department	*n* = 100 (*n* = 50 intervention vs. *n* = 50 control) patients with nasopharyngeal cancer	*Nutritional care planning/pathways* Nutritional management	31
Zhang 2020 [62] China	Pre-post study Radiation oncology department in public hospital (*n* = 1)	*n* = 50 pre- vs. *n* = 50 post- patients on chemo-radiotherapy with or at risk of developing cancer treatment-related oral mucositis	*Nutritional care planning/pathways* Nutritional interventions for patients with cancer treatment related oral mucositis	30
Zhang 2021 [33] China	Pre-post study Gastrointestinal surgery department in public and university-affiliated hospital (*n* = 1)	*n* = 60 (*n* = 30 pre- vs. *n* = 30 post-) patients with gastric cancer *n* = 10 nurses	*Nutritional care planning/pathways* Enteral nutrition	36.5

**Table 2:** Barriers.

TDF Domain	Barrier Descriptions	References *
Environmental context & resources	Lack of standardized, evidence-based nutritional protocols and guidelines	Atkins 2019 [36], Belluomini 2024 [37], Cook 2023 [43], Deftereos 2023 [44] ^, Ding 2023 [46], Gilbert 2021 [49], Levonyak 2021 [53], Paynter 2017 [58], Senesse 2017 [59], Wang 2014 [60], Zhang 2020 [62] ~, Zhang 2021 [33] ~
	Time demands associated with nutritional care delivery	Beck 2020 [26] *, Deftereos 2022 [27] ^, Garcia-Luna 2023 [48], Han 2018 [50], McCarter 2018 [29] *, Zhang 2021 [33] ~
	Insufficient staffing, resources, and multidisciplinary support for nutritional care	Deftereos 2022 [27] ^, Den 2021 [45], Findlay 2020 [28], Levonyak 2021 [53], McCarter 2018 [29] *, Naseer 2017 [31], Pasmann 2024 [57], Senesse 2017 [59], Zhang 2020 [62] ~
	Logistical challenges and inconvenience related to additional dietetic appointments	Beck 2020 [26] *
	Physical symptoms of cancer that hinder effective nutritional management	Beck 2020 [26] *
	Language barriers between staff nurses and patients during nutritional screening	Chen 2012 [42]
	Lack of clinician information and resources about nutritional care	McCarter 2018 [29] *, Pasmann 2024 [57]
	Lack of educational resources for cancer patients and caregivers about nutritional care	Wang 2014 [60]
	Poor quality of medical records regarding feeding information being provided to patients	Poveda 2018 [32]
	Administrative challenges in implementing nutritional care projects	Poveda 2018 [32]
	Structural characteristics of health services (e.g., shared care models, rural/regional settings)	Deftereos 2022 [27] ^
	Misalignment of existing workflows with nutritional care processes	Belluomini 2024 [37], Deftereos 2022 [27] ^
	Complexity and variability in patient care affecting consistent delivery of nutritional management	Deftereos 2022 [27] ^
Knowledge	Clinician lack of knowledge and/or awareness of nutritional care guidelines and procedures	Findlay 2020 [28], Gilbert 2021 [49], Han 2018 [50], McCarter 2018 [29] *, Zhang 2020 [62], Zhang 2021 [33] ~
	Patient and caregivers’ lack of nutrition knowledge	Paynter 2017 [58], Zhang 2020 [62] ~
	Patient and caregivers’ lack of awareness of available dietary counseling resources	Wang 2014 [60]
Skills	Lack of clinician skills for effective nutritional care assessment and management	Beck 2020 [26] *, Chen 2012 [42]
	Lack of performance monitoring tools for clinicians regarding nutritional education and care provision	Wang 2014 [60]
Intentions	Challenges in securing clinician commitment to participate in nutritional interventions	Poveda 2018 [32]
	Challenges in securing patient commitment to participate in nutritional interventions	Poveda 2018 [32], Zhang 2021 [33] ~
Beliefs about capabilities	Lack of clinician confidence to change patient dietary behaviours	Beck 2020 [26] *
Beliefs about consequences	Patients may not view nutrition as an important component of their cancer treatment	Beck 2020 [26] *
Motivation & goals	Cancer symptoms and premorbid issues reduce patients’ motivation to engage in nutritional care.	Beck 2020 [26] *, Paynter 2017 [58]
Emotion	Pre-existing emotional and psychological challenges can reduce a patient’s motivation and capacity to prioritise nutritional care during treatment	Beck 2020 [26] *
Social influences	Staff communication hierarchies and challenges in coordinating nutritional care	Deftereos 2022 [27] ^
Social/professional role and identity	Nurses are not empowered to make dietitian referral	Chen 2012 [42]
Memory, attention & decision processes	Complexity of dietetic guidelines	Findlay 2020 [28]

*, ~, ^ Identical symbols denote papers derived from the same study.

### 3.3. BCTs in Nutrition Interventions

**Figure 2:**
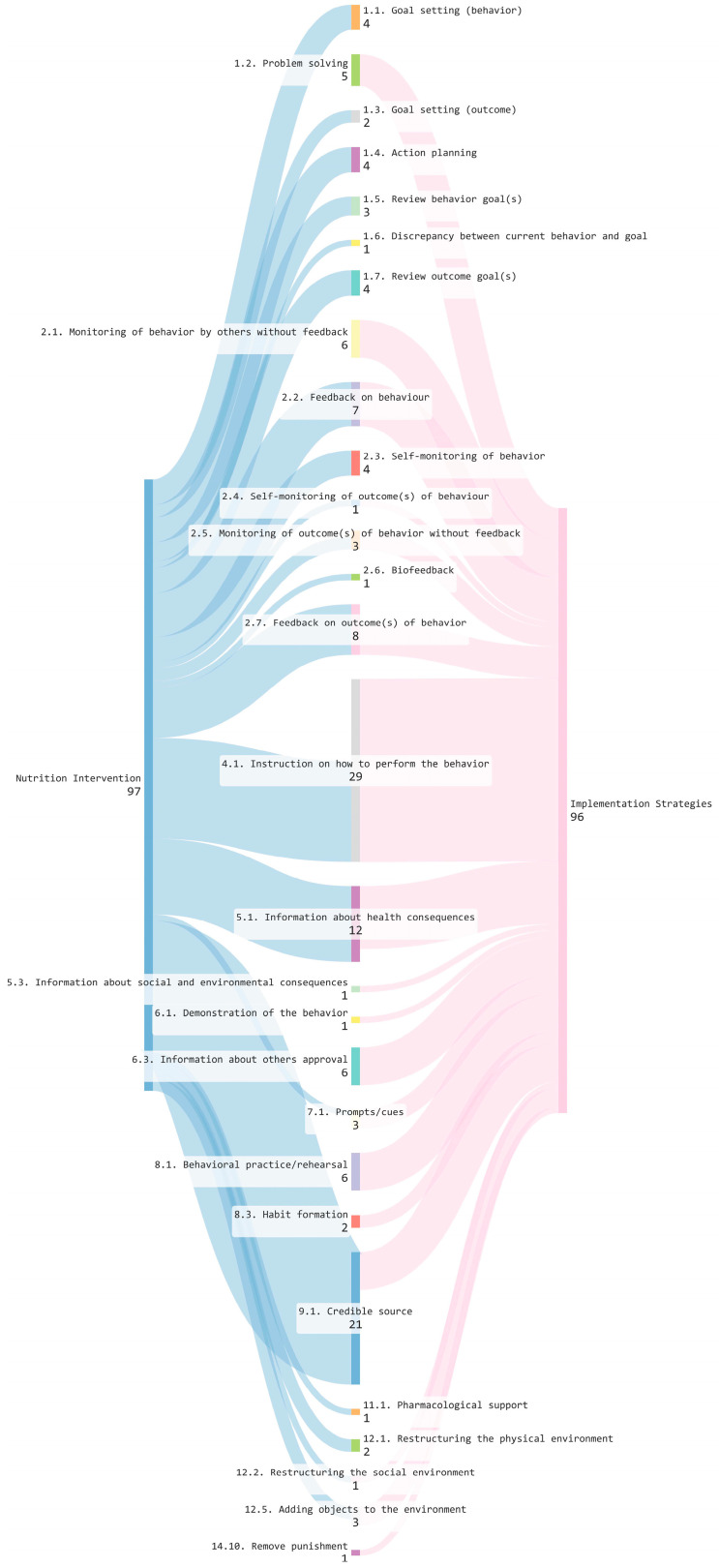
Sankey diagram of BCTs identified in nutrition interventions and implementation strategies.

A variety of BCTs targeting patients and HCPs were identified across the nutrition interventions (Table 3). Of the 31 unique studies reviewed, 19 (61%) incorporated at least 1 BCT within their intervention components. In total, 20 unique BCTs spanning 8 groups of the taxonomy were identified, reflecting a diverse yet recurring application of BCTs. Across all studies, 97 instances of BCT use were identified (Supplementary File S2). The spectrum of BCTs identified in nutrition interventions is illustrated in Figure 2.

BCTs embedded within nutrition interventions were mostly patient-targeted (*n* = 90; 93% instances). The most frequently identified BCTs (appearing more than 5 times) were ‘Credible source’ (*n* = 17; 18%), ‘Instruction on how to perform the behaviour’ (*n* = 13; 13%), ‘Information about health consequences’ (*n* = 9; 9%), ‘Feedback on outcome(s) of behaviour’ (*n* = 8; 8%), and ‘Feedback on behaviour’ (*n* = 7; 7%). These findings suggest the presence of a core group of BCTs that were consistently applied across interventions targeting patients.

Of the 7 (7%) instances specifically targeted HCPs, the interventions focused on nutritional support guidelines, nutritional care planning/pathways, and nutritional monitoring. These HCP-targeted BCTs were embedded within only a few interventions (*n* = 4, 13%). For example, in the nutritional support guidelines described by Naseer et al. [31], nursing education on mealtime targeted HCPs which incorporated the BCTs ‘Instruction on how to perform the behaviour’, ‘Information about the health consequences’, and ‘Credible source’. The BCT ‘Instruction on how to perform the behaviour’ was also identified in 2 nutritional care planning/pathways interventions, as reported by Poveda [32] and Zhang [33]. Additionally, a nutritional monitoring intervention described by Findlay et al. [28] incorporated ‘Feedback on outcome(s) of behaviour’ and ‘Prompts/cues’.

### 3.4. BCTs in Implementation Strategies

A range of BCTs targeting HCPs embedded with the corresponding implementation strategies that aimed to support adoption of the nutritional interventions were also identified (Table 4). Across 25 (66%) of the 38 included articles, 96 instances of BCT use were identified (Supplementary File S3), resulting in 18 unique BCTs targeting HCPs, spanning 10 groups of the taxonomy (Figure 2).

The most frequently observed BCT was ‘Instruction on how to perform the behaviour’ (*n* = 29; 30%), highlighting a strong emphasis on providing practical guidance to support implementation. This was followed by ‘Information about health consequences’ (*n* = 10; 10%), ‘Feedback on behaviour’ (*n* = 7; 7%), as well as ‘Monitoring of behaviour by others without feedback’, ‘Information about others’ approval’, ‘Behavioral practice/rehearsal’ and ‘Credible source’ (each *n* = 6; 6%). Other commonly observed BCTs included ‘Problem solving’ and ‘Feedback on outcome(s) of behaviour’ (each *n* = 5; 5%).

Less frequently observed techniques included ‘Monitoring of outcome(s) of behaviour without feedback’, ‘Prompts/cues’, and ‘Adding objects to the environment’ (each n = 3; 3%). Several BCTs were identified only once, such as ‘Remove punishment’, ‘Self-monitoring of outcome(s) of behaviour’, ‘Restructuring the social environment’, ‘Information about social and environmental consequences’ and ‘Demonstration of the behaviour’. Taken together, these findings indicate a predominant focus on instructional and informational strategies within implementation efforts by HCPs, with comparatively limited use of self-regulatory, social and environmental restructuring techniques.

### 3.5. BCTs Observed Alongside Positive Patient, Service, and Implementation Outcomes

#### 3.5.1. Intervention BCTs & Patient Outcomes

Twelve (39%) studies reported patient-level outcomes, including symptomatology (e.g., weight loss), patient satisfaction, functional measures (e.g., nutritional status, quality of life), and mortality. The distribution of BCTs observed across these categories is shown in Supplementary File S4. Among these, 2 BCTs were most frequently observed alongside positive outcomes (‘Instruction on how to perform the behaviour’ *n* = 12; ‘Credible source’ *n* = 9). These findings suggest that instructional clarity and source credibility within cancer care nutritional interventions may be critical in facilitating behaviour change that translates into measurable patient improvements.

#### 3.5.2. Implementation Strategy BCTs & Implementation and Service Outcomes

All 25 studies that applied BCTs as part of their implementation strategies reported on implementation and/or service outcomes. Commonly assessed domains included Fidelity (*n* = 18), Adoption (*n* = 16), Effectiveness (*n* = 14), Safety (*n* = 9), Feasibility (*n* = 7), and Timeliness (*n* = 6). A substantial majority (*n* = 21; 84%) documented positive findings across these domains, with the corresponding distribution of BCTs across categories presented in Supplementary File S5. The BCTs most often associated with positive implementation outcomes were also the ones more frequently used. In particular, ‘Instruction on how to perform the behaviour’ (*n* = 25) and ‘Information about health consequences’ (*n* = 9) appeared most frequently in studies with successful implementation outcomes. The prominence of these BCTs suggests the growing recognition of strategies that focus on both procedural guidance and contextual understanding for HCPs, to support the integration of nutritional care into clinical practice. A total of 22 (88%) studies reported outcomes related to Fidelity and/or Adoption. Of these, 19 studies reported positive outcomes, 2 studies reported neutral results [25,55] and 1 reported mixed outcomes [32]. ‘Instruction on how to perform the behaviour’ was the BCT most frequently observed alongside successful adoption (*n* =12) and fidelity (*n* = 12) (Supplementary File S5).

#### 3.5.3. Full Sequence of Change

Only 2 (6%) studies explicitly demonstrated a full sequence of change, in which improvements in implementation outcomes led to enhanced service delivery and ultimately better patient-level outcomes. These examples provide compelling case illustrations of how BCTs, when embedded across both intervention content and implementation processes, can drive holistic system improvement. In the study by Zhang et al. [33], enteral nutrition was implemented using 12 applications of 6 distinct BCTs. This effort resulted in sequential improvements in adoption and fidelity of healthcare delivery by HCPs, followed by gains in service-level outcomes including effectiveness, timeliness, and safety, culminating in improved patient functional status. Similarly, Findlay et al. [28] employed 9 instances of 5 BCTs, achieving implementation success in terms of appropriateness, acceptability, feasibility, and fidelity. These improvements were subsequently linked to increased service effectiveness and enhanced patient satisfaction.

### 3.6. TDF-BCT Linkages

Only BCTs derived from implementation strategies were analysed using the TDF-BCT mapping tool, as the barriers identified primarily related to implementation at the HCP level. This ensured alignment between the target population of the barriers and the corresponding BCTs. Alignment is illustrated in Table 4. Of the 19 articles that reported both implementation barriers that matched a TDF domain and strategies that represented BCTs, 13 (68%) aligned with existing mechanism mappings, 5 (26%) [36,37,43,44,57] produced evidence for potential hypothesised mechanisms not previously established (i.e., not identified in the Theory and Techniques Tool), and 1 (5%) diverged from known linkages [23], where established mechanisms did not result in implementation success. All 5 hypothesised mechanisms (observed with positive implementation and/or service outcomes) were associated with the Environmental Context and Resources domain, addressed by BCTs included ‘Instruction on how to perform the behaviour’ (*n* = 4 instances), ‘Feedback on outcome(s) of behaviour’ (*n* = 2 instances), ‘Information about health consequences’ (*n* = 2 instances), ‘Credible source’ (*n* = 2 instances), and 1 instance each for ‘Monitoring of behaviour by others without feedback’, ‘Information about others approval’, and ‘Problem solving’.

## 4. Discussion

This review identified a wide range of BCTs used within oncology nutritional care, both in intervention content and in implementation strategies. Despite this diversity, a core group of BCTs consistently emerged across studies. For intervention content, commonly used techniques included ‘Credible source’, ‘Instruction on how to perform the behaviour’, and ‘Information about health consequences’. Similarly, implementation strategies frequently incorporated ‘Instruction on how to perform the behaviour’, ‘Information about health consequences’, and ‘Feedback on behaviour’. These techniques were frequently present in nutrition interventions with favourable patient outcomes, and in implementation strategies with favourable implementation or service outcomes, a pattern that underscores their central role in effective intervention design and delivery. QuADS assessments indicated moderate to high methodological quality across all included studies. While the total scores suggest most studies met a substantial proportion of QuADS criteria, cut-offs were based on those applied in previously published systematic reviews [63]. Due to the lack of official guidance, these user-defined thresholds are for convenience and are not empirically validated. They should therefore be considered pragmatic descriptors rather than formal indicators of study quality, consistent with the tool’s design for item-level appraisal rather than categorical classification [16]. Nonetheless, the predominance of studies meeting high-quality criteria support the credibility of the findings of this review.

Barriers to implementing nutrition interventions were most identified in relation to the ‘Environmental Context and Resources’ domain of the TDF. While this review focused on individual-level barriers, it is likely that broader system-level constraints exert a substantial influence on implementation success. These structural challenges may be more comprehensively captured through the application of multi-level frameworks such as the updated Consolidated Framework for Implementation Research (CFIR 2.0) [64], which offers a detailed lens for understanding constraints experienced by individuals [15], particularly within the Environmental Context and Resources domain.

Many of the implementation efforts engaged HCPs in addition to patients. This dual targeting is particularly important when nutrition interventions are aligned with clinical guidelines or best practice standards, which require active participation from both those delivering and receiving care. However, only 10 studies, approximately one-third, applied BCTs to both populations, suggesting that opportunities remain to better integrate patient and provider-focused strategies.

The emergence of ‘Instruction on how to perform the behaviour’ across both patient and HCP groups suggests that practical guidance may be a foundational component of effective nutritional care in cancer settings, but may be underutilised in current practice. Similarly, ‘Credible source’ was frequently linked with positive patient outcomes, highlighting the importance of trusted messengers in enhancing patient engagement, adherence, and clinical outcomes. Compared to BCTs identified in smoking cessation interventions [65], such as prompting commitment, social award, and identify associated techniques, the BCTs observed in nutrition interventions appear contextually distinct. This reinforces the need for context-specific understanding of the application of BCTs. Our findings suggest that future interventions should explicitly emphasise endorsement from credible authorities, such as dietitians, oncologists, and national cancer organisations, to reinforce the benefits and importance of nutritional care.

For HCPs, both ‘Instruction on how to perform the behaviour’ and ‘Information about health consequences’ appear to be particularly influential in supporting the adoption of nutrition interventions. These findings suggest that HCPs may be more likely to integrate nutrition into their practice when they receive both procedural training and have a clear understanding of the clinical rationale. This reinforces the need for implementation strategies that build skills and further HCP education about the value of nutritional care. Compared to a previous coding study of BCTs in implementation strategies for diabetes care [66], the difference in frequently identified BCTs underscores the context-specific nature of BCT application. This variation suggests that the selection and effectiveness of BCTs are influenced by the unique clinical characteristics of different conditions, despite the development of BCT taxonomies intended to be applicable across a range of behavioural domains (e.g., healthy eating, smoking, physical activity) [21]. In addition, it is important to recognise that dietetics and nutrition are distinct specialised disciplines, and limited resourcing within healthcare systems often results in the dissemination of nutrition-related information being delegated to non-dietetic HCPs, such as oncologists. This redistribution of responsibility points to the need for not only enhancing general HCP capability but also encourages interdisciplinary collaboration and resource allocation to support specialist input.

In contrast, the absence of BCTs related to Social support, Reward and threat, Identity, Self-belief, and Covert learning in nutrition interventions and their implementation strategies may reflect a tendency to prioritise more tangible and practical techniques over those involving deeper psychological or sustained social engagement [67]. These underutilised BCTs may hold untapped potential for enhancing engagement and sustained behaviour change, particularly in nutrition care within cancer care. This omission highlights a need for broader exploration and integration of diverse BCTs in future nutrition intervention and implementation strategy design, supported by more inclusive frameworks that enable their application and evaluation.

The identification of positive sequential changes in a small number of studies further illustrates how the integrated application of BCTs within both nutrition interventions and implementation strategies can help align implementation adoption (or alternatively its antecedents) and fidelity, with clinical effectiveness and eventually clinical outcomes and patient experience. This is particularly salient for complex health interventions such as nutritional care that require coordinated, multi-level change. Despite retrospective coding efforts, such positive sequential change was identified in only 2 studies. This low yield signals an urgent need for more systematic and robust implementation planning and data collection and reporting. Without these improvements, the field is unlikely to keep pace with the rapid scientific progress and may continue to fall short in uncovering the underlying patterns of implementation success.

The emergence of potential novel mechanisms suggests that existing TDF-BCT mapping may not fully capture the breadth of BCTs that could potentially address implementation barriers faced by healthcare providers, particularly those rooted in environmental context and resources. This again calls for more systematic approaches, such as integrating implementation frameworks (e.g., CFIR 2.0) to better identify, target, and accumulate evidence on mechanisms of change and ultimately to advance the field of implementation science. In the 2 studies that demonstrated a full sequence of change from implementation outcomes through to service and patient outcomes, a similar set of barriers was reported, primarily concerning the lack of clinician knowledge regarding nutritional care guidelines and time constraints. The implementation strategies employed in response largely focused on instructing clinicians on how to perform the desired behaviour, often delivered by credible sources or accompanied by peer endorsement. These findings can be interpreted through the lens of Kazdin’s criteria for establishing mechanisms of change [68]. Kazdin outlined 7 key criteria, i.e., strong association, specificity, consistency, experimental manipulation, timeline, gradient, and plausibility/coherence, that determines whether a proposed mechanism is truly causal [69]. In both above example studies, several of these criteria might have been partially fulfilled. While these studies did not explicitly articulate mechanisms of action, they provide early evidence that could be used to formulate novel mechanisms for testing in future research. Further mapping the strategies onto specific behavioural determinants (e.g., knowledge, environmental context and resources), identifying potential moderators (e.g., clinician baseline knowledge) and necessary preconditions for activating mechanisms (e.g., access to training materials, leadership buy-in) would enable a more rigorous identification of causal pathways that lead to proximal outcomes (e.g., clinician behaviour change) and distal outcomes (e.g., patient nutritional status) [70]. Although a formal causal mediation analysis was beyond the scope of the current review, this could be an important next step in advancing implementation science in the context of nutrition care.

The review has several strengths. It employed a comprehensive literature search, systematic data extraction, and rigorous coding using established implementation science frameworks. The use of the TDF-BCT mapping tool enabled a structured analysis of real-world BCT application in addressing implementation barriers, to assess the alignment with known mechanisms of behaviour change, while also identifying novel linkages, particularly in relation to the ‘Environmental Context and Resources’ domain. The involvement of a multidisciplinary team with expertise in both clinical nutrition and implementation science added depth and relevance to this evidence synthesis.

Despite its strengths, this review has some limitations that should be acknowledged. The patterns identified between BCTs and outcomes were observational in nature, limiting the ability to draw causal inferences. The effectiveness of individual BCTs or specific combinations was not experimentally evaluated, and as such, conclusions about their relative impact remain uncertain. A further limitation is the reliance on retrospective coding of published descriptions to theoretical frameworks, which is constrained by the completeness and clarity of reporting. As a result, while the review describes the frequency of reported BCTs, it was not possible to assess the quality, intensity, or functional role of each BCT within interventions, nor to distinguish whether BCTs were actively delivered, nominally included, or simply mentioned. In addition, implementation and service outcomes were often reported qualitatively, with limited use of standardised measures. This variability in reporting may have influenced the comparability of findings across studies.

Further research to explore and evaluate the effectiveness of specific BCTs or combinations to better identify the active ingredients that drive successful implementation and improved patient outcomes would be useful. It is equally important to investigate the contextual factors that influence the uptake, integration, and sustainability of nutrition interventions in oncology, as these factors are likely to vary across settings and populations. Notably, only around 60% of included articles reported implementation barriers faced by HCPs, and patient perspectives were largely absent. Addressing this gap by capturing a broader range of interest holder experiences will help to develop effective interventions and strategies. Collectively, these efforts will support the expanded and tailored application of BCTs across interest holder groups to enhance engagement, effectiveness, and sustainability of nutritional care in oncology.

## 5. Conclusions

This review provides a comprehensive synthesis of BCTs employed in nutrition interventions and their implementation within oncology care. While a wide range of BCTs were identified, a core group focused on practical guidance, trusted communication, and contextual understanding which consistently emerged as being frequently aligned with positive outcomes across patient, service, and implementation levels. For clinical practice, more explicit specifications of BCTs may facilitate improved integration of nutrition interventions in oncology care. Future research may help evaluate the effectiveness of specific individual BCTs and their combinations across both interventions and approaches to their implementation. Additionally, greater attention to contextual factors that influence implementation, inclusion of diverse interest holder perspectives, particularly those of patients and caregivers, and explicit adherence to reporting guidelines and implementation and behavioural frameworks would support more systematic accumulation of implementation evidence. Taken together, more rigorous implementation planning and standardised data collection and reporting are needed to enable robust testing of nutrition interventions and implementation strategies and to determine the underlying mechanisms that drive implementation success.

## Figures and Tables

**Table 3:** BCTs in Nutrition Interventions.

Author, Year	Intervention Component(s)	BCTs Identified	Patient Outcomes
Adriaans 2022 [35]	Provide information on recommended nutritional schedule; Weight and nutrition diary for self-monitoring; Email communication with dietitian; Dietitian reviews diary before consultation	1.5. Review behaviour goal(s) 1.7. Review outcome goal(s) 2.3. Self-monitoring of behaviour 2.4. Self-monitoring of outcome(s) of behaviour 4.1. Instruction on how to perform the behaviour 9.1. Credible source	Symptomatology Satisfaction Function
Atkins 2019 [36]	Nutritional follow-up to support oral feeding and stop artificial nutrition	2.2. Feedback on behaviour 2.6. Biofeedback 2.7. Feedback on outcome(s) of behaviour 9.1. Credible source	None reported
Beck 2020 [26] Beck 2021 [25] Britton 2019 [39] Britton 2024 [40] McCarter 2018 [29] Murray 2019 [30]	Motivational interviewing to enhance adherence; Written nutrition plan with daily checklist	1.1. Goal setting (behaviour) 1.3. Goal setting (outcome) 1.4. Action planning 1.5. Review behaviour goal(s) 1.6. Discrepancy between current behaviour and goal 1.7. Review outcome goal(s) 2.2. Feedback on behaviour 2.3. Self-monitoring of behaviour 2.7. Feedback on outcome(s) of behaviour 4.1. Instruction on how to perform the behaviour 5.1. Information about health consequences 12.5. Adding objects to the environment	Satisfaction Symptomatology Function Mortality
Belluomini 2024 [37]	Education on importance of nutrition in cancer; Referral of at-risk patients to dietetics service	5.1. Information about health consequences 9.1. Credible source	None reported
Blake 2022 [38]	Early dietetic intervention for knowledge and symptom management; Referral to local dietitian and speech pathologist post-treatment	1.7. Review outcome goal(s) 2.7. Feedback on outcome(s) of behaviour 4.1. Instruction on how to perform the behaviour 5.1. Information about health consequences 9.1. Credible source	Symptomatology
Carr 2022 [41]	Written handouts and recovery log for patients; Dietitian contacts patient within 48 h of discharge and monitors regularly	1.1. Goal setting (behaviour) 1.5. Review behaviour goal(s) 1.7. Review outcome goal(s) 2.3. Self-monitoring of behaviour 12.5. Adding objects to the environment	Symptomatology Function Mortality
Chen 2012 [42]	Action plans for malnutrition risk	1.4. Action planning	None reported
Deftereos 2023 [44] Deftereos 2022 [27]	Nutritional counselling with symptom management and ongoing review	2.7. Feedback on outcome(s) of behaviour 5.1. Information about health consequences	Symptomatology
Den 2021 [45]	Individual dietary counselling; Education on expected nutrition impact symptoms and post-surgery nutrition; Post-discharge follow-up	1.1. Goal setting (behaviour) 2.2. Feedback on behaviour 2.7. Feedback on outcome(s) of behaviour 5.1. Information about health consequences 9.1. Credible source	None reported
Ding 2023 [46]	Individualized energy and meal targets with weekly goals; Clinicians and nurses monitor and adjust nutrition plan	1.3. Goal setting (outcome) 2.2. Feedback on behaviour 2.7. Feedback on outcome(s) of behaviour	Function
Findlay 2020 [28]	Pre-treatment clinic for assessment, education and counselling; Nutrition Care Dashboard integrated into MDT meetings (Targeting HCPs)	2.7. Feedback on outcome(s) of behaviour 4.1. Instruction on how to perform the behaviour 7.1. Prompts/cues 12.2. Restructuring the social environment	Symptomatology Satisfaction
Kiss 2019 [51]	Nutrition assistants reinforce diet information	5.1. Information about health consequences 9.1. Credible source	Symptomatology
Moore 2021 [56]	Preoperative nutritional assessment and education; Oral Nutritional Supplements log; Feeding regimen reviewed and optimized; Second nutritional assessment pre-surgery	2.2. Feedback on behaviour 2.3. Self-monitoring of behaviour 4.1. Instruction on how to perform the behaviour 5.1. Information about health consequences 9.1. Credible source	Symptomatology
Naseer 2017 [31]	Nursing staff ensure pleasant eating environment; Minimize unnecessary interventions during mealtimes; Nursing education on mealtime (Targeting HCPs)	4.1. Instruction on how to perform the behaviour 5.1. Information about health consequences 9.1. Credible source 12.1. Restructuring the physical environment	Satisfaction
Paynter 2017 [58]	Patient instructions on supplements and immune-nutrition	4.1. Instruction on how to perform the behaviour 5.1. Information about health consequences 9.1. Credible source	Symptomatology Satisfaction
Poveda 2018 [32]	Discharge planning for home enteral feeding; Training patients/carers on enteral feeding device; Written information on feeding regimen and troubleshooting; Regular follow-up for feeding regimen review Discharge protocol for patients requiring home enteral feeding (Targeting HCPs)	1.4. Action planning 2.2. Feedback on behaviour 2.7. Feedback on outcome(s) of behaviour 4.1. Instruction on how to perform the behaviour 9.1. Credible source	Satisfaction
Senesse 2017 [59]	Nutritional counselling by dietitians	9.1. Credible source	None reported
Wang 2014 [60]	Education for patients and caregivers on malnutrition prevention; Dietary counselling before chemotherapy;	4.1. Instruction on how to perform the behaviour 5.1. Information about health consequences 9.1. Credible source	None reported
Zhang 2021 [33]	Nutritional status assessed after admission and 1 week after enteral nutrition; Nurse records enteral nutrition start time, energy intake, and underfeeding; Encourage functional exercise (e.g., chewing gum, abdominal massage); Feeding intolerance prophylaxis management; Early enteral nutrition health education and communication strategy; Nurse education on preventing underfeeding in enteral nutrition (Targeting HCPs)	2.5. Monitoring of outcome(s) of behaviour without feedback 4.1. Instruction on how to perform the behaviour 5.1. Information about health consequences 9.1. Credible source 11.1. Pharmacological support	Function

**Table 4:** BCTs targeting HCPs (in Implementation Strategies).

Author, Year	TDF Domain	Implementation Strategy	BCTs Identified	Implementation/ Service Outcomes	Evidence Alignment
Atkins 2019 [36]	1. Environmental context and resources	Assess clinician compliance at three time points	2.1. Monitoring of behaviour by others without feedback	Adoption Fidelity Efficiency	Suggests hypothesised mechanism
Beck 2020 [26]	1. Skills 2. Beliefs about Capabilities 3. Environmental Context & resources	Training workshop with education, role play, and feedback; One-day clinical shadowing for real-time feedback	2.2. Feedback on behaviour 4.1. Instruction on how to perform the behaviour 6.1. Demonstration of the behaviour 8.1. Behavioural practice/rehearsal	Fidelity Feasibility	Aligns with existing mapping
Beck 2021 [25]	1. Skills 2. Beliefs about capabilities 3. Environmental context & resources	Two-day EAT training workshop; One-day clinical shadowing for real-time feedback; Follow-up booster workshop and shadowing	2.2. Feedback on behaviour 4.1. Instruction on how to perform the behaviour	Fidelity	Diverges from existing linkages
Belluomini 2024 [37]	1. Environmental context and resources	Thoracic oncologists were trained by a skilled dietitian to implement the Assess, Advise, and Refer (AAR) process into clinical practice	4.1. Instruction on how to perform the behaviour	AdoptionEffectiveness	Suggests hypothesised mechanism
Britton 2019 [39]	N/A	Trainers travelled to each hospital to provide training; Academic detailing through shadowing; Provide performance feedback to managers; Use visual prompts for EAT principles	2.2. Feedback on behaviour 2.7. Feedback on outcome(s) of behaviour 4.1. Instruction on how to perform the behaviour 7.1. Prompts/cues 12.5. Adding objects to the environment	Fidelity Cost Feasibility Safety	N/A
Chen 2012 [42]	1. Skills;2. Environmental context and resources	Educate nurses on nutritional screening and simplified tool;Audit compliance with best practice screening;Establish project team with nurse leaders;One-to-one engagement and reinforcement with nurses;Secure protected time for audits	4.1. Instruction on how to perform the behaviour2.2. Feedback on behaviour6.3. Information about others approval8.1. Behavioural practice/rehearsal14.10. Remove punishment	AcceptabilityAdoptionFidelityTimeliness	Aligns with existing mapping
Cook 2023 [43]	1. Environmental context and resources	Pre- and post- implementation service evaluation;	2.7. Feedback on outcome(s) of behaviour	Effectiveness Safety	Suggests hypothesised mechanism
Deftereos 2023 [44]	1. Environmental context and resources	Train site dietitians on pathway and processes	4.1. Instruction on how to perform the behaviour 5.1. Information about health consequences	Adoption Feasibility Fidelity Safety Timeliness	Suggests hypothesised mechanism
Ettori 2019 [47]	1. Environmental context and resources 2. Memory, attention & decision processes	Implement computer-assisted decision support system (CDSS)	12.5. Adding objects to the environment	Fidelity Effectiveness Safety	Aligns with existing mapping
Findlay 2020 [28]	1. Environmental context and resources 2. Memory, attention & decision processes 3. Knowledge	Develop evidence-based nutrition care pathway; Educate dietitians on PG-SGA tool use; Use Nutrition Care Dashboard for feedback and audit; Provide staff education and support for intervention delivery; Engage senior opinion leaders in implementation	2.7. Feedback on outcome(s) of behaviour 4.1. Instruction on how to perform the behaviour 6.3. Information about others approval	Fidelity Cost Feasibility Acceptability Appropriateness Effectiveness	Aligns with existing mapping
Gilbert 2021 [49]	1. Environmental context and resources 2. Knowledge	Outreach geriatric team provides training and advice	4.1. Instruction on how to perform the behaviour 5.1. Information about health consequences	Adoption Fidelity Safety Effectiveness	Aligns with existing mapping
Han 2018 [50]	1. Environmental context and resources 2. Knowledge	Reemphasize screening policy via memo and SOP; Circulate dietitian referral procedures; Conduct audit and implement remedial measures	2.2. Feedback on behaviour 2.7. Feedback on outcome(s) of behaviour 4.1. Instruction on how to perform the behaviour 8.1. Behavioural practice/rehearsal 8.3. Habit formation	Adoption Fidelity Effectiveness	Aligns with existing mapping
Kiss 2019 [51]	N/A	Follow the Standards of Quality Improvement Reporting Excellence (SQUIRE 2.0); Develop eight-week training module for nutrition assistants	2.1. Monitoring of behaviour by others without feedback 2.5. Monitoring of outcome(s) of behaviour without feedback 4.1. Instruction on how to perform the behaviour	Appropriateness Feasibility Effectiveness Timeliness	N/A
Krznaric 2019 [34]	N/A	Promote guidelines at conferences and distribute full text	4.1. Instruction on how to perform the behaviour 9.1. Credible source	Acceptability Fidelity Effectiveness Timeliness Safety	N/A
Ladna 2025 [52]	N/A	Create smart set for EPI diagnosis and treatment	7.1. Prompts/cues	Adoption Effectiveness	N/A
Levonyak 2021 [53]	1. Environmental context and resources 2. Knowledge 3. Skills	Conduct multidisciplinary meetings on MST use	4.1. Instruction on how to perform the behaviour 5.1. Information about health consequences 9.1. Credible source	Adoption, Sustainability Effectiveness Safety	Aligns with existing mapping
Martin-McGill 2020 [55]	N/A	Provide patients and caregivers with dietary education and resources	4.1. Instruction on how to perform the behaviour 5.1. Information about health consequences 12.5. Adding objects to the environment	Adoption Feasibility	N/A
McCarter 2018 [29]	1. Knowledge 2. Environmental context and resources	Obtain executive endorsement for implementation; Train dietitians in screening tools and provide booster sessions; Clinical psychologists shadow dietitians to resolve barriers; Provide site performance feedback via reports and calls; Supply nutrition and depression screening tools during training	1.2. Problem solving 2.1. Monitoring of behaviour by others without feedback 2.5. Monitoring of outcome(s) of behaviour without feedback 4.1. Instruction on how to perform the behaviour 6.3. Information about others approval 8.1. Behavioural practice/rehearsal 9.1. Credible source	Adoption Fidelity	Aligns with existing mapping
Naseer 2017 [31]	1. Environmental context and resources 2. Knowledge 3. Skills	Form project team with nurse leaders and influencers; Use emails and texts for team communication; Present audit results and identify barriers with nurses; Analyse barriers and develop improvement plan via JBI-GRIP; Include project in staff orientation and preceptor training; Engage doctors to maintain protected mealtimes	1.2. Problem solving 2.1. Monitoring of behaviour by others without feedback 4.1. Instruction on how to perform the behaviour 5.1. Information about health consequences 6.3. Information about others approval	Adoption Fidelity Sustainability	Aligns with existing mapping
Pasmann 2024 [57]	1. Environmental context and resources	Letter of support provided by Utah Cancer Specialists; Pre-implementation education session on nutrition, MST use, and change principles; Second education session on identifying oral nutritional supplement samples; Pre- and post-knowledge quizzes for RNs and APPs to assess learning	1.2. Problem solving 2.7. Feedback on outcome(s) of behaviour 4.1. Instruction on how to perform the behaviour 5.1. Information about health consequences 6.3. Information about others approval 9.1. Credible source	Adoption Fidelity Feasibility Effectiveness	Suggests hypothesised mechanism
Poveda 2018 [32]	1. Intentions 2. Environmental context and resources	Team leader provided training, developed tools, collected data, and supervised implementation; Multidisciplinary meetings to explain project goals and obtain authorization	2.1. Monitoring of behaviour by others without feedback 6.3. Information about others approval	Adoption Acceptability, Fidelity	Aligns with existing mapping
Wang 2014 [60]	1. Environmental context and resources 2. Skills 3. Knowledge	Education sessions for nurses on malnutrition and prevention; nurses certified after passing test; Develop and distribution educational material for GI cancer patients and caregivers, including BMI check tool; Improve Hospital Information System for doctors and nurses	2.4. Self-monitoring of outcome(s) of behaviour 4.1. Instruction on how to perform the behaviour 5.1. Information about health consequences 7.1. Prompts/cues 9.1. Credible source	Adoption Fidelity Effectiveness Timeliness Effectiveness	Aligns with existing mapping
Zeng 2023 [61]	N/A	Apply PDCA cycle for continuous improvement in nutrition management; Weekly checks of nutrition plan completion, diet records, nutritional status, and oral mucositis	1.2. Problem solving 2.1. Monitoring of behaviour by others without feedback 2.5. Monitoring of outcome(s) of behaviour without feedback	Safety	N/A
Zhang 2020 [62]	1. Environmental context and resources 2. Knowledge	Establish multidisciplinary team with dietitian; Develop and deliver staff training/education programs; Nurse-led provision of nutritional education to patients	4.1. Instruction on how to perform the behaviour 12.2. Restructuring the social environment	Adoption Fidelity Effectiveness	Aligns with existing mapping
Zhang 2021 [33]	1. Environmental context and resources 2. Knowledge 3. Intentions	Conduct nurse training on enteral nutrition underfeeding prevention and management; certify with knowledge test; Develop standardised functional exercise protocol; share via social media and provide on-site guidance; Compile enteral nutrition education manual; share via instant messaging app and reinforce with face-to-face education	4.1. Instruction on how to perform the behaviour 5.1. Information about health consequences 5.3. Information about social and environmental consequences	Adoption Fidelity Effectiveness Timeliness Safety	Aligns with existing mapping

## Data Availability

Not applicable.

## Supplementary Materials

The following supporting information can be downloaded at: https://www.mdpi.com/article/10.3390/nu18020242/s1, Supplementary File S1: PRISMA Checklist; Supplementary File S2: Behaviour Change Techniques within nutrition interventions components; Supplementary File S3: Behaviour Change Techniques in implementation strategies and their links to the Theoretical Domain Framework; Supplementary File S4: Behaviour Change Techniques identified in implementation strategies that aligned with positive patient outcomes; Supplementary File S5: Behaviour Change Techniques identified in implementation strategies that aligned with positive implementation and service outcomes.

## Abbreviations

The following abbreviations are used in this manuscript:

BCT	Behaviour Change Technique
CFIR 2.0	updated Consolidated Framework for Implementation Research
HCP	Healthcare Professional
QuADS	Quality Assessment with Diverse Studies
TDF	Theoretical Domains Framework
