# Supplementary material for: Identifying Behaviour Change Techniques in Cancer Nutrition Interventions and Their Implementation Contexts: A Systematic Review
Source: Nutrients. 2026 Jan 12;18(2):242. doi: 10.3390/nu18020242 (PMC12845379; doi:10.3390/nu18020242)
Supplement: Supplementary file 1 [file nutrients-18-00242-s001.zip › Supplementary file S5.pdf]

Supplementary file S5. Behaviour Change Techniques identified in implementation strategies that aligned with positive implementation and service outcomes.

BCT	Acceptability	Appropriateness	Adoption	Fidelity	Effectiveness	Safety	Feasibility	Timeliness	Efficiency	Cost	Sustainability
1.2. Problem solving	-	-	3	3	1	1	1	-	-	-	1
2.1. Monitoring of behaviour by others without feedback	-	1	4	3	-	1	1	-	1	-	1
2.2. Feedback on behaviour	1	-	2	4	-	1	1	1	-	1	-
2.4. Self-monitoring of outcome(s) of behaviour	-	-	1	1	1	-	-	1	-	-	-
2.5. Monitoring of outcome(s) of behaviour without feedback	-	1	1	1	-	1	1	-	-	-	-
2.7. Feedback on outcome(s) of behaviour	1	1	2	4	3	2	3	-	-	2	-
4.1. Instruction on how to perform the behaviour	3	2	12	12	9	5	5	5	-	3	2
5.1. Information about health consequences	-	-	7	6	5	3	2	3	-	-	2
5.3. Information about social and environmental consequences	-	-	1	1	1	1	-	1	-	-	-
6.1. Demonstration of the behaviour	-	-	-	1	-	-	-	-	-	-	-
6.3. Information about others approval	2	1	5	5	2	-	2	1	-	1	1
7.1. Prompts/cues	-	-	2	2	2	1	1	2	-	1	-
8.1. Behavioural practice/rehearsal	1	-	3	4	-	-	-	1	-	-	-
8.3. Habit formation	-	-	1	1	-	-	-	-	-	-	-
9.1. Credible source	1	-	4	3	4	1	1	2	-	-	1
12.2. Restructuring the social environment	-	-	1	1	1	-	-	-	-	-	-
12.5. Adding objects to the environment	-	-	-	2	1	1	1	-	-	1	-
14.10. Remove punishment	1	-	1	1	-	-	-	1	-	-	-
