# Supplementary material for: Identifying Behaviour Change Techniques in Cancer Nutrition Interventions and Their Implementation Contexts: A Systematic Review
Source: Nutrients. 2026 Jan 12;18(2):242. doi: 10.3390/nu18020242 (PMC12845379; doi:10.3390/nu18020242)
Supplement: Supplementary file 1 [file nutrients-18-00242-s001.zip › Supplementary file S2.pdf]

Supplementary file S2. Behaviour Change Techniques within nutrition interventions components

Author, year	Intervention component	BCTs identified	Patient Outcomes
Adriaans 2022 (43)	An informative component in which patients had access to information on the recommended nutritional schedule, i.e., the recommended number of calories and amount of protein.	4.1. Instruction on how to perform the behaviour<br>9.1. Credible source	**Symptomatology:** No significant difference in absolute weight change from baseline and 12 weeks between the intervention and usual care groups (P=0.085). Post-hoc analysis, excluding patients who did not use the intervention more than once, showed significantly less weight loss over time in the intervention group compared to usual care (p = 0.048). No difference in symptom scores.<br><br>**Satisfaction:** No significant difference in patient satisfaction in hospital care.<br><br>**Function:** No difference in health-related quality of life (HRQoL) scores.
	A self-management component, consisting of a weight and nutritional diary, to monitor their weight, calorie, and protein intake.	2.3. Self-monitoring of behaviour<br>2.4. Self-monitoring of outcome(s) of behaviour	
	An email component, for direct communication with the dietitian.	9.1. Credible source	
	A follow-up component. Before every consultation, the dietitian was able to review the weight and nutritional diary and use the information in subsequent contact with the patient.	1.5. Review behaviour goal(s)<br>1.7. Review outcome goal(s)	
Atkins 2019 (32)	Nutritional follow-up to facilitate resumption of oral feeding and withdrawal from artificial nutrition. Included weekly determination of body weight, clinical surveillance of hydration status and evaluation of oral food intake.	2.2. Feedback on behaviour<br>2.6. Biofeedback<br>2.7. Feedback on outcome(s) of behaviour<br>9.1. Credible source	None reported

Beck 2020 (21)<br>Beck 2021 (20)<br>Britton 2019 (45)<br>Britton 2024 (46)<br>McCarter 2018 (24)<br>Murray 2019 (25)	Use of motivational interviewing (eliciting patient motivation for undergoing treatment, highlighting relationship between nutritional status and treatment outcomes; exploring (in)consistency between patient behaviour, motivation to survive, nutritional status and requirements for maintaining an adequate nutritional status; and developing a concrete plan).	1.1. Goal setting (behaviour)<br>1.4. Action planning<br>1.6. Discrepancy between current behaviour and goal<br>5.1. Information about health consequences	**Satisfaction:** Patient-rated therapeutic alliance lower after training in EAT intervention, but difference was not statistically significant (95% CI = -6.76, 3.55; p = 0.542).<br><br>**Symptomatology:** EAT intervention associated with less weight loss, lower depression scores, and improved QoL, also fewer/shorter unplanned hospital admissions (not statistically significant).<br><br>**Function:** Intervention patients' nutritional status was significantly better than controls (nutritional scores: b=-1.53, p=0.03).<br><br>**Mortality:** There were 64 deaths in the 5 years (1826 days) after enrolment, 36/151 (24%) in the control group and 28/157 (18%) in the intervention group. Adjusted logistic regression showed reduced odds of mortality in 5 years following radiotherapy in the intervention group compared to the control group (OR, 0.33; 95% CI, 0.11-0.96; P=.04), with an absolute risk reduction of 17% (95% CI,
	Use of a written nutrition plan to document and reinforce patient goals, including an objective measure of nutritional status. Patients work through a daily list of behaviours and tick off each item as it was completed. Patients receive ongoing follow-up by a dietitian for at least 6 weeks after treatment.	1.1. Goal setting (behaviour)<br>1.3. Goal setting (outcome)<br>1.4. Action planning<br>1.5. Review behaviour goal(s)<br>1.7. Review outcome goal(s)<br>2.2. Feedback on behaviour<br>2.7. Feedback on outcome(s) of behaviour<br>2.3. Self-monitoring of behaviour<br>4.1. Instruction on how to perform the behaviour<br>12.5. Adding objects to the environment	

			0.01-0.33; P=.03), a relative risk reduction of 55% (95% CI, 0.22-0.92; P=.04), and an number needed to treat of 6 (95%CI, 4-13). Kaplan-Meier survival curves produced an unadjusted 5-year actuarial survival rate of 76% (0.68-0.82) for the control group and 82% (0.75-0.87) for intervention group (p=.22). Cox proportional hazards regression found lower 5-year mortality risk in the intervention compared to control group (hazard ratio, 0.39; 95%CI, 0.16-0.96; P=.04).
Belluomini 2024 (33)	Advise patients about the importance of nutrition in cancer.	5.1. Information about health consequences	None reported
	Refer patients at nutritional risk to the dedicated dietetic service.	9.1. Credible source	
Blake 2022 (44)	Early dietetic intervention to e.g., enhance patient's knowledge and understanding about maintaining nutritional status and weight, potential negative impact of malnutrition, symptom management strategies.	4.1. Instruction on how to perform the behaviour<br>5.1. Information about health consequences<br>9.1. Credible source	**Symptomatology:** Post-implementation cohort lost 1.2% less weight (p=0.34) and had reduced nutritional status decline (p=0.53) compared with pre-implementation group. The research team states this was a clinically important outcome despite being non-significant. Critical weight loss remained high in each group.
	On treatment completion, patients were referred to their local dietitian and speech pathologist for ongoing rehabilitation as clinically indicated, with EN ceased when a patient's oral intake was deemed adequate.	1.7. Review outcome goal(s)<br>2.7. Feedback on outcome(s) of behaviour<br>9.1. Credible source	

Carr 2021 (47)	Patients given written handouts describing what to expect, as well as an activity and recovery log consisting of daily checklists that outline specific postoperative goals for each day.	1.1. Goal setting (behaviour)<br>2.3. Self-monitoring of behaviour<br>12.5. Adding objects to the environment	**Symptomatology:** In post-protocol group less post-operative weight loss (p=0.002)<br><br>**Function:** Significant reductions in length of hospital stay (p <0.001) in post-protocol group.<br><br>**Mortality:** In-hospital mortality and 30-day mortality, did not differ between groups (p=0.22).
	The outpatient dietitian contacts the patient within 48 h of discharge and monitors the patient every 2-3 days by phone.	1.5. Review behaviour goal(s)<br>1.7. Review outcome goal(s)	
Chen 2012 (48)	Initiation of appropriate action plans when at-risk patients for malnutrition are identified.	1.4. Action planning	None reported
Deftereos 2023 (35)<br>Deftereos 2022 (22)	Nutritional counselling (including information about importance of adequate nutrition) +/- symptom management and ongoing review.	2.7. Feedback on outcome(s) of behaviour<br>5.1. Information about health consequences	**Symptomatology:** No difference between groups for preoperative weight change. There was a non-significant reduction in malnutrition in the intervention group between baseline (65%) and surgery (48%).
Den 2021 (49)	Individual dietary counselling.	1.1. Goal setting (behaviour)<br>9.1. Credible source	None reported
	Education on expected nutrition impact symptoms and nutrition post surgery.	5.1. Information about health consequences<br>9.1. Credible source	
	Post-discharge patient follow up.	2.2. Feedback on behaviour<br>2.7. Feedback on outcome(s) of behaviour<br>9.1. Credible source	

Ding 2023 (50)	Based on nutritional assessment, individualized energy intake and meal targets were determined for each patient, with achievable weekly targets set for nutritional outcomes (e.g. laboratory parameters, changes in body weight).	1.3. Goal setting (outcome)	**Function:** Patients in personalised groups had more rapid improvements in hand grip strength (p<0.01), self-care ability (p<0.01), serum albumin concentration (p<0.01) after surgery.
	Clinicians and nurses monitored the patients' implementation of the nutritional prescriptions by assessing indicators of nutritional status and dynamically adjusting nutritional plan as required.	2.2. Feedback on behaviour<br>2.7. Feedback on outcome(s) of behaviour	
Findlay 2020 (23)	Supportive Care-Led Pre-Treatment Clinic delivered structured, targeted pre-treatment assessment, intervention, education and counselling to patients and caregivers.	4.1. Instruction on how to perform the behaviour<br>12.2. Restructuring the social environment	**Symptomatology**: No statistically significant difference in BMI or mean weight change between 2 groups however mean weight change remained below the clinically important threshold poor prognosis threshold of ≥ 5% at all but one point for patients on new model of care. Nutritionally related risks were identified in 74% of patients. 24 of 34.<br><br>**Satisfaction:** Statistically significant improvement in all dimensions of patient satisfaction scores (p = 0.03 for overall satisfaction, p<0.01 for patient-perceived benefit, p=0.02 for
	Targeting HCPs<br><br>Nutrition Care Dashboard highlighting nutrition outcome data integrated into MDT meetings.	2.7. Feedback on outcome(s) of behaviour<br>7.1. Prompts/cues	

			dietitian/nutrition-assistant interpersonal
Kiss 2019 (55)	Nutrition assistants provided education and reinforced diet information previously provided by the dietitian.	5.1. Information about health consequences<br>9.1. Credible source	**Symptomatology:** No significant difference in mean percentage weight change over duration of treatment.
Moore 2021 (59)	Patients underwent a preoperative nutritional assessment with dedicated HNC dietitian and receives nutritional education as well as disclosed details of nutritional intake and relevant clinical and lifestyle information.	4.1. Instruction on how to perform the behaviour<br>5.1. Information about health consequences<br>9.1. Credible source	**Symptomatology:** Modified Nutrition-Related Index (mNRI) measure of nutritional status was significantly higher in post-protocol (n=16) cohort compared to pre-protocol (n=7) cohort (105 vs 89.5, p = 0.03).<br>For patients enrolled in ERAS protocol, there's a strong negative correlation between modified Nutrition-Related Index (mNRI) and number of complications (P = 0.01), specifically, fistula rate (P = 0.04) and unplanned reoperation (P = 0.04).
	Patients were provided an Oral Nutritional Supplements data-sheet to record consumption.	2.3. Self-monitoring of behaviour	
	Patients' feeding regimen was reviewed and optimised.	2.2. Feedback on behaviour	
	A second nutritional assessment was conducted with the dietitian 1 week before surgery via telephone that assessed compliance with ONS, tolerance, and continued nutritional education.	2.2. Feedback on behaviour<br>4.1. Instruction on how to perform the behaviour<br>9.1. Credible source	
Naseer 2017 (26)	Nursing staff ensured a pleasant eating environment is provided without clutter in over-way and immediate areas.	12.1. Restructuring the physical environment	**Satisfaction:** Patient satisfaction survey ‘excellent’ rating improved from 2% to 20%. Improved eating environment. Patients felt more cared for. Less clinical interventions during mealtimes.
	Unnecessary clinical interventions were minimised during mealtimes.	12.1. Restructuring the physical environment	

	Targeting HCPs<br><br>Nursing staff received education on mealtime care, including its importance to hematology-oncology patients.	4.1. Instruction on how to perform the behaviour<br>5.1. Information about health consequences<br>9.1. Credible source	
Paynter 2017 (60)	Patients received instructions via an information leaflet and by nursing staff about consuming supplements. The importance of pre-operative immune-nutrition for post-operative recovery was explained.	4.1. Instruction on how to perform the behaviour<br>5.1. Information about health consequences<br>9.1. Credible source	**Symptomatology:** Number of days until patients received full diets post-operatively were 0.80 days shorter post-implementation (not statistically significant).<br><br>**Satisfaction:** 41% (n=7) of patients informed (via verbal and/or written information) of the reasons to take immuno-nutrition supplements claimed the immuno-nutrition to have been beneficial to their health, such as leading to a good recovery after surgery or increasing their energy levels. Two-thirds of patients claimed that the immuno-nutrition did not affect their appetite (n=11).
Poveda 2018 (27)	Discharge planning for patients who will continue to require enteral feeding at home, initiated by the multidisciplinary healthcare team from the outset of admission.	1.4. Action planning	**Satisfaction:** Education given to caregivers accepted positively.
	Patients and/or their carers received adequate training on the use and care of enteral feeding device prior to discharge.	4.1. Instruction on how to perform the behaviour<br>9.1. Credible source	

	Patients and/or their carers were provided with written information about the feeding regimen, pump instructions, dislodged tube information and relevant contact numbers prior to discharge.	4.1. Instruction on how to perform the behaviour<br>9.1. Credible source	
	Patients have regular follow-up appointments with relevant health practitioners for review of their feeding regimen and tube use/care.	2.2. Feedback on behaviour<br>2.7. Feedback on outcome(s) of behaviour<br>9.1. Credible source	
	Targeting HCPs<br><br>The healthcare team had access to a discharge protocol for patients requiring home enteral feeding.	4.1. Instruction on how to perform the behaviour	
Senesse 2017 (61)	Provision of nutritional counselling by dieticians.	9.1. Credible source	None reported
Wang 2014 (62)	Patients and caregivers were educated about malnutrition and prevention approaches.	4.1. Instruction on how to perform the behaviour<br>5.1. Information about health consequences<br>9.1. Credible source	None reported
	A dietary counselling session was conducted for patients at risk of malnutrition before chemotherapy.	4.1. Instruction on how to perform the behaviour<br>5.1. Information about health consequences<br>9.1. Credible source	
Zhang 2021 (28)	Patients were assessed for nutritional status after admission and 1 week after EN by the nurse or dietitian.	2.5. Monitoring of outcome(s) of behaviour without feedback	**Function:** Preoperative PGSGA scores were not significantly different between the baseline audit

	The nurse records the time EN was started after surgery, the daily energy intake, and the incidence of underfeeding.	2.5. Monitoring of outcome(s) of behaviour without feedback	and post-implementation audit. Postoperative PGSGA scores were lower post-implementation (12.9 ± 1.5) compared with the baseline audit (14.0 ± 1.8) (p=0.013).
	Patients were encouraged and guided to perform functional exercise, including chewing gum and abdominal massage 1 day after surgery.	4.1. Instruction on how to perform the behaviour	
	Patients received feeding intolerance prophylaxis management.	11.1. Pharmacological support	
	The nurse conducts early EN health education for the patient, informs them about the benefits and precautions to be taken for EN, and establishes a non-verbal communication strategy.	4.1. Instruction on how to perform the behaviour<br>5.1. Information about health consequences<br>9.1. Credible source	
	Targeting HCPs<br><br>The nurse receives education on how to prevent underfeeding in EN.	4.1. Instruction on how to perform the behaviour	
