# Supplementary material for: Identifying Behaviour Change Techniques in Cancer Nutrition Interventions and Their Implementation Contexts: A Systematic Review
Source: Nutrients. 2026 Jan 12;18(2):242. doi: 10.3390/nu18020242 (PMC12845379; doi:10.3390/nu18020242)
Supplement: Supplementary file 1 [file nutrients-18-00242-s001.zip › Supplementary file S3.pdf]

Supplementary file S3. Behaviour Change Techniques in implementation strategies and their links to the Theoretical Domain Framework

Author, year	TDF Domain	Implementation strategy	BCTs identified	Mechanistic link (T&T tool)	Implementation/Service Outcomes	Evidence Alignment
Atkins 2019 (32)	1. Environmental context and resources	Assessment of clinician compliance at three time points	2.1. Monitoring of behaviour by others without feedback	No evidence	**Adoption, Fidelity:** High compliance with delivery of nutritional care according to the care pathway, with compliance rates over 80% for the majority of components. The highest compliance was for completion of nutrition assessment on admission, initiation of parenteral nutrition and post-discharge follow-up. The lowest were for enteral nutrition commencement, nutrition assessment on discharge, and frequency of review post day +2 of HSCT. Mean compliance across all components and all audit periods was 84%.<br><br>**Efficiency:** the implementation of the care pathway was delivered without the need for additional allocation of dietetic or other health professional resources.	Suggests emerging mechanism

Beck 2020 (21)	1. Skills<br>2. Beliefs about Capabilities<br>3. Environmental Context & resources	Training workshop, which included (i) information provision (regarding the background, rationale, principles, and strategies) and (ii) modelling, role play, self-reflection, and peer and trainer video feedback of intervention principles and strategies.	2.2. Feedback on behaviour<br>4.1. Instruction on how to perform the behaviour<br>6.1. Demonstration of the behaviour<br>8.1. Behavioural practice/rehearsal	LINKS (Sk:4.1)<br>LINKS (Sk:8.1)<br>LINKS (BaCa:4.1)<br>LINKS (BaCa:6.1)<br>LINKS (BaCa:8.1)	**Fidelity:** Statistically significant improvement in the application of the EAT Intervention after training of dietitians compared to pre-training treatment as usual consultations. Achievement of adherence and competency: benchmarks varied between elements measured, some met high fidelity (>80%) others didn't (<80%). E.g. information provided on relationship between malnutrition and radiotherapy outcomes doubled in intervention group. increase in motivational and behavioural strategies employed during consultations in intervention group. written	Aligns with existing mapping

		Training followed by one-day clinical "shadowing", whereby the trainers accompanied dietitians onsite during usual practice to observe and provide "real-time" troubleshooting and feedback		LINKS (Sk:8.1) LINKS (BaCa:8.1)	nutrition planner more likely in intervention sessions. non-unique intervention features of EAT intervention e.g. nutritional assessment occurred equally in intervention and TAU groups.<br><br>**Feasibility:** The number of and duration of dietetic consultations did not differ between control and intervention group (training)	
Beck 2021 (20)	1. Skills 2. Beliefs about capabilities 3. Environmental context & resources	Training in EAT comprised a 2-day workshop	4.1. Instruction on how to perform the behaviour	LINKS (Sk:4.1) LINKS (BaCa:4.1)	**Fidelity:** Variable fidelity of intervention delivery. Dietitians' adherence to intervention delivery ranged from 13 to 96% for each checklist item. The mean overall Behaviour Change Counselling Index practitioner score was 23.17, meaning that on average, each dietitian used behaviour change counselling 'to some extent'. The average dietitian score on the CTS-R interpersonal effectiveness item was in the 'expert' range.	Diverges from existing linkages
		Provision of 1-day clinical 'shadowing'	2.2. Feedback on behaviour	No evidence		
		Provision of a follow-up 'booster' workshop (comprising 1-day workshop, and 1-day shadowing).	2.2. Feedback on behaviour 4.1. Instruction on how to perform the behaviour	LINKS (Sk:4.1) LINKS (BaCa:4.1)		

Belluomini 2024 (33)	1. Environmental context and resources	Thoracic oncologists were trained by a skilled dietitian to implement the Assess, Advise, and Refer (AAR) process into clinical practice	4.1. Instruction on how to perform the behaviour	None	**Adoption:** between July 2022 and Feb 2023, 40% of patients scheduled for a first oncological evaluation received nutritional screening from oncologist using a validated tool (<30% of oncologists check nutritional status in Italy, and only 16% use a validated tool). All the screened patients were advised about the importance of nutritional aspects during cancer care. VS. pre-implementation: 8 patients with thoracic malignancies were referred to the dietitian upon their request between Nov 2021 and Jun 2022. Of these patients, only 2 resulted at nutritional risk at the first visit with the dietitian.<br><br>**Effectiveness:** 7 patients were detected at nutritional risk and referred to the dietetic service	Suggests emerging mechanism
Britton 2019 (45)	N/A	Trainers travelled to each hospital and provided an initial 3 days of training, which was supported by a "booster" training session 2 months later.	4.1. Instruction on how to perform the behaviour	N/A	**Fidelity:** Dietitians delivered the intervention satisfactorily to prespecified levels<br><br>**Cost, Feasibility:** Low ongoing cost of intervention (after initial training) as no additional physical/personnel resources required	N/A

		Provision of academic detailing through shadowing days,	2.2. Feedback on behaviour	N/A	**Safety:** Fewer radiotherapy interruptions (8% intervention, 14% control). Fewer/shorter unplanned hospital admissions (not statistically significant)	
		Using intervention performance - which was relayed back to managers	2.7. Feedback on outcome(s) of behaviour	N/A		
		Providing tools/resources eg. stickers and water bottles prompting EAT principles	4.1. Instruction on how to perform the behaviour<br>7.1. Prompts/cues<br>12.5. Adding objects to the environment	N/A		
Chen 2012 (48)	1. Skills;<br>2. Environmental context and resources	Education provided to registered nurses regarding the best available evidence on nutritional screening, and dietitian introduced a validated simplified version of the screening tool.	4.1. Instruction on how to perform the behaviour	LINKS (Sk:4.1)	**Acceptability:** Most of the nurses felt that the nutritional screening tool was easy to use and straightforward to follow.<br><br>**Adoption, Fidelity:** Pre- vs. Post-implementation, a validated screening tool is used, 100% vs. 100%; Patients are screened upon admission, 96% vs. 100%<br><br>**Timeliness:** a significant improvement in appropriate action plans are initiated when at-risk	Aligns with existing mapping

		Monitor audit compliance with best practice criteria for nutritional screening	2.2. Feedback on behaviour	None	patients for malnutrition are identified, from 46% to 100% (x2 = 22.967; P = 0.000), indicating a timely and relevant dietician referral	
		Establishment of the project team that included two Nurse Managers, two Nurse Clinicians and one Nurse Educator.	6.3. Information about others approval	None		
		One-to-one engagement and constant reinforcement with the individual registered nurse.	8.1. Behavioural practice/rehearsal	LINKS (Sk:8.1)		

		The team had requested the Nurse Manager to provide them with a protected time, which was the overlapping shift and allowed time for the team to conduct the audit without compromising the patients' care	14.10. Remove punishment	N/A		

Cook 2023 (34)	1. Environmental context and resources	A pre- and post-implementation service evaluation was conducted to assess the impact of these changes. Results presented at stakeholder meeting, a multidisciplinary team developed an updated dietetic-led pre-RIG risk assessment.	2.7. Feedback on outcome(s) of behaviour	None	**Effectiveness:** 46.7% (n=7) in pre-implementation group and 16.7% (n=3) in post-implementation group required 2 wks pre-RIG NGT feeding. If pre-implementation risk assessment had been used on post-implementation group an additional 50% (n=9) pts would have required 2 weeks NGT feeding.<br><br>**Safety:** Implementation of updated pre-RIG insertion risk assessment avoided 2 weeks of inpatient NGT feeding for 50% (n=9) of post-implementation group, without an increase in pt complications. Reduced insertion delays and costs relating to NGT feeding.	Suggests emerging mechanism

Deftereos 2023 (35)	1. Environmental context and resources	Site study dietitians participated in training to ensure familiarity with the pathway and study processes.	4.1. Instruction on how to perform the behaviour<br>5.1. Information about health consequences	None	**Adoption:** Compared with the control group, there was a significant increase in overall, outpatient and phone dietetics contacts before surgery. However, inpatient contacts remained the same. Control vs. Intervention<br>- Seen by dietitian before surgery, n (%) 19 (55) 35 (100) $p < 0.001$<br>- Total dietetics contacts, mean ± SDb 2.2 ± 3.7 5.9 ± 3.9 $p < 0.001$<br>- Outpatient contacts, mean ± SDb 1.0 ± 2.7 3.7 ± 2.7 $p < 0.001$<br>- Phone contacts, mean ± SDb 0.1 ± 0.3 1.0 ± 1.3 $p < 0.001$<br>- Inpatient contacts, mean ± SDb 1.1 ± 1.9 1.4 ± 3.0 $p = 0.56$<br><br>**Feasibility:** Recruitment rate was 81%. Although the withdrawal rate was high at 34%, this was the result of a change in treatment, rather than due to study-related factors.<br><br>**Fidelity:** Screening within 1 week of diagnosis in the multidisciplinary meeting occurred in 91% of patients. The pathway follow-up schedule was followed 74% of the time, and dietitians were able to prescribe the	Suggests emerging mechanism

					interventions as per the pathway on 84% of occasions.<br><br>**Safety:** Non-statistically significant decreases in preoperative nutrition-related hospital admissions, and surgical complications were demonstrated in patients who underwent neoadjuvant therapy. No reported adverse events associated with the study.<br><br>**Timeliness:** Received dietetics contact within 1 week of diagnosis, n (%) 11 (31) 27 (77) p < 0.001	

Ettori 2019 (51)	1. Environmental context and resources<br>2. Memory, attention & decision processes	Implementation of a computer assisted decision support system (CDSS) to supervise a nutritional intervention by MDT to increase guideline adherence and outcomes	12.5. Adding objects to the environment	LINKS (ECR:12.5)	**Fidelity:** Using CDSS allowed increased rate of days in compliance with calorie and protein targets in after grp by 50%. However, overall compliance rates were low and only reached 30% after intervention.<br><br>**Effectiveness:** Increase in use of Enteral Nutrition in after grp. Rate of Parenteral Nutrition increased to 76%.<br><br>**Safety:** ICU-acquired infection and lengths of stay were similar in the two groups	Aligns with existing mapping

Findlay 2020 (23)	1. Environmental context and resources<br>2. Memory, attention & decision processes<br>3. Knowledge	evidence-based nutrition care pathway with clearly defined screening and assessment protocols, timing and frequency of dietetic contact before, during and after treatment and routine use of validated malnutrition screening and nutrition assessment tools.	4.1. Instruction on how to perform the behaviour	LINKS (Kn:4.1)	**Fidelity**: For the Supportive Care-Led Pre-Treatment Clinic,97% (n = 33 of 34) of eligible patients were offered a clinic appointment and 100% of these attended. For the Nutrition Care Dashboard integrated into the weekly radiotherapy list, discussion occurred as intended at 100% of MDT meetings during the intervention period whilst the MDT clinical year was in session.<br><br>**Cost**: New model of care avoided 3.92 unplanned admissions and related costs of $AUD121K per annum.<br><br>**Feasibility, Acceptability, Appropriateness**: Focus groups confirmed clear support at the multidisciplinary team level for continuing the new model of care	Aligns with existing mapping
		Dietitians were provided with education and support regarding use of the PG-SGA tool	4.1. Instruction on how to perform the behaviour	LINKS (Kn:4.1)		

		The Nutrition Care Dashboard fulfilled the dual role of communicating timely clinical nutrition outcome information broadly to the MDT but also provided the function of ongoing weekly audit and feedback through highlighting whether adherence to agreed benchmarks were being met.	2.7. Feedback on outcome(s) of behaviour	None	**Effectiveness:** Post-implementation data (n = 34) demonstrated improved process and clinical outcomes: pre-treatment dietitian assessment; use of a validated nutrition assessment tool before, during and after treatment. Patients receiving the new model of care were significantly more likely to complete prescribed radiotherapy and systemic therapy.	
		Staff given education and support to deliver intervention	4.1. Instruction on how to perform the behaviour	LINKS (Kn:4.1)		

		Senior opinion leaders within the participating cancer centre organization were invited to join the team of investigators.	6.3. Information about others approval	None		

Gilbert 2021 (53)	1. Environmental context and resources<br>2. Knowledge	An outreach geriatric team (geriatrician and a dietitian) visited each centre to provide training and advice to the health professionals focused on nutritional screening and management of undernutrition in accordance with prevailing guidelines. This was done through scheduled meetings with the staff, regular visits to the wards, and the dissemination of written, informative documents to both patients and professionals.	4.1. Instruction on how to perform the behaviour<br>5.1. Information about health consequences	LINKS (Kn:4.1)<br>LINKS (Kn:5.1)	**Adoption, Fidelity**: Increase in guideline-consistent clinical management from 1.4% of patients in the control group to 39.2% of patients in the intervention group (p = 0.0002).<br><br>**Safety:** Adverse events were more frequently reported in the intervention condition, in which 58% of patients had at least one adverse event reported (p = 0.0500). The majority of these events<br>were of low to moderate severity in both groups and were generally less severe in the intervention group (p = 0.0109) (Table S4). Of note, none of these events were considered to be related to the study.<br><br>**Effectiveness:** Evaluation of nutritional status was significantly more accurate in the intervention condition compared to control (p < 0.0001) but remained imperfect (49% of accurate nutritional assessments in the intervention phase compared to 18% in control);<br>All patients in the intervention	Aligns with existing mapping

					group were assessed using at least three of the four diagnosis elements (BMI, weight loss, albumin assay, and MNA), with over 80% having all four available in the medical records. In the control phase, 29% of patients had two<br>elements, almost half (49%) had three, and 15% had all four. BMI was calculated in 100% of patients in both groups;<br>BMI measured (100% vs 100%), serum albumin measured (71.2% vs 85.1%, p = 0.0411), weight loss measured (82.2% vs. 98.6%, p = 0.0007), MNA performed (19.2 vs 97.3%, p < 0.0001);<br>The anorexia scale was documented in 92% of patients in the intervention group versus less than 3% in the control group (p < 0.0001).<br>Adequate diagnosis of nutritional status 13 (17.8%) 36 (48.6%) <0.0001;<br>Prescriptions in accordance with nutritional status 2 3 (4.1) 31 (41.9) <0.0001	

Han 2018 (54)	1. Environmental context and resources<br>2. Knowledge	Reemphasis of the nutritional screening policy in specialist clinics by written memo and circulation of standard operating procedure for nutrition screening process.	4.1. Instruction on how to perform the behaviour<br>8.1. Behavioural practice/rehearsal<br>8.3. Habit formation	LINKS (Kn:4.1)	**Adoption, Fidelity:** significantly improved screening rate using MST from 6.3% to 79.5% between the initial and re-audit phases ($p<0.001$)<br><br>**Effectiveness:** no parallel significant improvement with the dietitian referral rate. 14.2% of ≤2 were referred to dietitians in the initial phase, the rate was 15.9% during the re-audit phase	Aligns with existing mapping
		Circulation of dietitian's referral procedure for nurses and physician reference	8.1. Behavioural practice/rehearsal<br>8.3. Habit formation	None		
		Conduct audit to determine the rate of adherence to nutritional screening, and to implement remedial measures for improved patient care.	2.2. Feedback on behaviour<br>2.7. Feedback on outcome(s) of behaviour	None		

Kiss 2019 (55)	N/A	Followed the Standards of Quality Improvement Reporting Excellence (SQUIRE 2.0), and conducted prospective pre- and post-test data collection	2.1. Monitoring of behaviour by others without feedback<br>2.5. Monitoring of outcome(s) of behaviour without feedback	N/A	**Appropriateness, Feasibility**: the low proportion of same day referrals back to the dietitian indicated this was both a feasible and appropriate use of the NA role<br><br>**Effectiveness:** the proportion of dietitian time spent with high-risk patients did not differ among the groups. (Pre-implementation, 88% of dietitian time was spent with	N/A

		An eight-week training module was developed to upskill two NAs knowledge of the operation of the head and neck cancer clinic, use of the screening tool and triggers for referral back to the dietitian.	4.1. Instruction on how to perform the behaviour	N/A	high-risk patients, 11% with intermediate risk and 1% with low risk patients. Post-implementation 86% of dietitian time was spent with high-risk patients, 12% with intermediate risk and 2% with low-risk patients.<br><br>**Timeliness:** The time to the commencement of feeding through the nasogastric tube did not differ between the pre- and post-implementation cohorts (33 days, SD 10 vs 31 days, SD 10, respectively, P = 0.24).<br>Time to commencement of feeding through the PEG tube was longer in the post-implementation cohort compared to the pre-implementation cohort but did not reach statistical significance (13.7 days, SD 13 vs 7.1 days, SD 7.8, respectively, P = 0.95).	

Krznaric 2019 (29)	N/A	During the whole period of follow-up from the time of publishing guidelines constant promotional activities at Croatian congresses and meetings of Croatian Medical Societies were performed, and the full text of guidelines was distributed to all of the oncologists by post.	4.1. Instruction on how to perform the behaviour<br>9.1. Credible source	N/A	**Acceptability:** The percentage of oncologists that are familiar with Croatian guidelines remained high (did not show statistically significant difference in the follow up period 2011-2018) 73.4% (2011) vs 81.8% (2013) vs 78.2% (2018)<br><br>**Fidelity:** 54% of oncologists prescribe nutritional support to 50% or less of their patients, and almost third of oncologists prescribe EPA in insufficient quantity.<br><br>**Effectiveness**: 80% of oncologists believe that the Croatian national guidelines changed their approach in treating patients with CC syndrome; the use of metabolic modulators remained at high level<br><br>**Timeliness**: Oncologists mostly recommend patients to use nutritional support during 1 year or more (43%, N = 49), or 2mo to 1 year (42%, N = 48)<br><br>**Safety:** Incidence of adverse effects at the recommended doses of EPA is low; MA can have	N/A

					possible adverse effects that may appear during usage	

Ladna 2025 (56)	N/A	A smart set was developed to address gaps in EPI diagnosis and treatment, providing prespecified PERT dosages and orders for key tests, referrals, and smoking cessation support.	7.1. Prompts/cues	N/A	**Adoption:** The BPA was triggered a total of 30,838 times over the 24 months immediately after its implementation. Only 624 (2.02%) of these triggers resulted in the smart set being opened. Over a period of 24 months, there was a consistent use of the smart set without the need for re-education. However there was a slight decrease in the monthly use of the smart set over a period of 24 months after initial implementation with a slope of negative 0.2016.<br><br>**Effectiveness:** A statistically significant increase in the proportion of patients on a minimum therapeutic dose of PERT from 61.9% to 72.9% (OR 1.64, P < .001) was observed. Ordering of pancreatic elastase, A1c, vitamin D, and DEXA increased from 20.4% to 29.9% (OR 1.67, P < .001), 54.7%–62.1% (OR 1.36, P ¼ .001), 30.9%–48.1% (OR 2.06, P < .001) and 10%–18% (OR 1.96,	N/A

					(P < .001), respectively after initiation of BPA and smart set. An increase in vitamin D supplementation from 25.5% to 33.4% (OR 1.45, P < .001) and an increase in the proportion of patients with known metabolic bone disease status from 13.2% to 23.3% (OR 1.99, P < .001) was also observed.	

Levonyak 2021 (57)	1. Environmental context and resources<br>2. Knowledge<br>3. Skills	Conducted multidisciplinary meetings with all clinic nursing staff, physicians, and administrators to demonstrate the use of the MST and the importance of identifying patients with malnutrition.	4.1. Instruction on how to perform the behaviour<br>5.1. Information about health consequences<br>9.1. Credible source	LINKS (Kn:4.1)<br>LINKS (Kn:5.1)<br>LINKS (Sk:4.1)	**Adoption, Sustainability:** The percentage of patients with GI cancer with documented assessment by the registered dietitian increased from 5.1% in October 2018 prior to our interventions to 21.8% in July 2019 and has sustained in the 15%-20% range thereafter.<br><br>**Effectiveness:** Overall referrals to registered dietitian has increased in number<br><br>**Safety:** A nonsignificant decrease in serum albumin level between initial visit and 3-month follow-up: –0.01 g/dL (CI, –0.19 to 10.16; P 5 .84).<br>During the 3-month follow-up, 26/63 (41.2%) patients had one or more emergency department visits, 16/63 (25.3%) had at least one hospital admission, and only 6/63 (9.5%) had greater than one hospital admission.	Aligns with existing mapping

Martin-McGill 2020 (31)	N/A	Pts and caregivers were given dietary education from a dietitian and were provided with 7 day meal plans, recipes, dietary information sheets and food diaries.	4.1. Instruction on how to perform the behaviour<br>5.1. Information about health consequences<br>12.5. Adding objects to the environment	N/A	**Adoption:** Recruitment rate of 28.6% (12/42) of eligible population.<br><br>**Feasibility**: Retention was poor; only four of 12 patients completed the three-month diet.	N/A
McCarter 2018 (24)	1. Knowledge<br>2. Environmental context and resources	Senior trial investigators solicited the support and endorsement of executive staff from each site for implementation of the intervention and recommendations based on clinical guidelines	6.3. Information about others approval	None	**Adoption, Fidelity:** The clinical practice change strategy significantly improved the odds of implementation of four of the six guideline recommendations ($p < .05$).<br>Post vs. Pre:<br>Guideline 1: Dietitian contact weekly during RT 71.5% vs 63.5%, $p = .0339$;<br>Guideline 2: Dietitian contact fortnightly for 6 weeks post-RT 47.7% vs 48.6%, $p = .7686$;	Aligns with existing mapping

		Dietitians were trained in the administration of screening tools and were asked to screen all patients using such tools. A booster training session was conducted to trouble shoot any issues arisen with implementation of the behaviour change intervention or practice guideline recommendations.	1.2. Problem solving<br>4.1. Instruction on how to perform the behaviour<br>9.1. Credible source	LINKS (Kn:4.1)<br>LINKS (Sk:4.1)	Guideline 3: Nutritional assessment at Week 1 of RT 89.7% vs 69.1%, p = .0002;<br>Guideline 4: Monitor weight, intake, and nutritional status during and after RT 88.8% vs 56.7%, p<.0001;<br>Guideline 5: Depression screening at Week 1<br>of RT 81.3% vs 0.7%, p<.0001;<br>Guideline 6: Depression referral at Week 1 of RT 42.1% vs 0.0%, p = .0537	

		Clinical psychologists attended the radiotherapy department dietetic clinics to 'shadow' dietitians, and provided advice, feedback, and support to resolve implementation barriers including systems changes to facilitate regular appointments.	1.2. Problem solving	None		
		Feedback on site performance relative to agreed benchmarks was provided in written reports and during telephone contacts every 3-4 months to the head of the dietetics departments	2.1. Monitoring of behaviour by others without feedback<br>2.5. Monitoring of outcome(s) of behaviour without feedback	None		

		Services and staff had access to nutrition assessment and depression-screening tools that were provided during training, so as to facilitate discussion and practice	8.1. Behavioural practice/rehearsal	LINKS (Sk:8.1)		
Naseer 2017 (26)	1. Environmental context and resources<br>2. Knowledge<br>3. Skills	Established a project team, including nurse manager and senior staff nurse, based on their expertise and positive attitudes and ability to reach and influence other nurses. The team identified a group of senior staff who could give support and endorse this project.	6.3. Information about others approval	None	**Adoption, Fidelity:** The post implementation audit results showed nurses ensuring a pleasant eating environment that was free of clutter (Criterion 1) achieved 93%, showing a significant improvement of 50% compliance. Nurses received education on mealtime care (Criterion 2) maintained at 100%, and assessment of patients' ability to eat on admission (Criterion 3) also achieved 100% compliance, with an increase of 5% from the baseline results. The results showed a decrease of 3% compliance for unnecessary clinical	Aligns with existing mapping

		The team leaders introduced the project to the members, highlighting the importance of recommended practice, audits, and the Gantt chart to guide the project timeline. The members and stakeholders used emails and hand phone text messages for communication.	2.1. Monitoring of behaviour by others without feedback<br>5.1. Information about health consequences	LINKS (Kn:5.1)	interventions during mealtime (Criterion 4).<br><br>**Sustainability:** The post implementation results at six months showed audit results being sustained at 100% compliance in all criteria.	

		The team adopted the bottom-up approach and presented the pre-implementation audit and patient satisfaction survey results to the ward nurses, and together they identified barriers that caused the under-achieved targets of the criteria from the baseline results. The team also gathered nurses' feedback and encouraged them to ask questions and suggest strategies.	1.2. Problem solving	None		

		The team entered the identified barriers into JBI-GRIP, analysed and developed an improvement plan, conducted several rounds of meetings with the nurses to disseminate the improvement plan, which was disseminated via several rounds of meetings.	4.1. Instruction on how to perform the behaviour	LINKS (Kn:4.1) LINKS (Sk:4.1)		

		To include this project as part of the orientation package for new staff. Clinical preceptors to educate new staff on the importance of mealtime assistance. Existing staff nurses to be role models to new staff in providing mealtime assistance to patients.	4.1. Instruction on how to perform the behaviour	LINKS (Kn:4.1) LINKS (Sk:4.1)		

		The team leader conducted engagement sessions with the doctors to increase their awareness of the importance of maintaining a 'protected mealtime' for patients and asked them for help in re-scheduling their patient-contact time.	4.1. Instruction on how to perform the behaviour<br>5.1. Information about health consequences	LINKS (Kn:4.1)<br>LINKS (Sk:4.1)<br>LINKS (Kn:5.1)		
Pasmann 2024 (36)	1. Environmental context and resources	A letter of support for the project was provided by Utah Cancer Specialists	6.3. Information about others approval	No evidence	**Adoption, Fidelity:** Overall, 101 unique patients were identified to be screened across 12 weeks, and the rate of adherence to the	Suggests emerging mechanism

		Prior to nutritional screening implementation, clinical staff received education about cancer-related nutritional issues and using the MST. The education session consisted of a 30-minute narrated slideshow presentation discussing nutrition and its effect on clinical outcomes, the project's inclusion and exclusion criteria, how to use the MST, and elements of leading change	1.2. Problem solving<br>4.1. Instruction on how to perform the behaviour<br>5.1. Information about health consequences<br>9.1. Credible source	None	screening process during the project period was 69%.<br><br>**Feasibility:** Based on the results of this project, the implementation of a standardized nurse-led nutritional screening process can identify patients receiving oncology care who are at risk for malnutrition.<br><br>**Effectiveness:** Of the 80 patients who received an initial screening, 45 were identified as having an MST score of 1 or greater, with 30 scoring 2 or greater, which indicated nutritional risk from project onset. After receiving education, RNs and APPs exhibited an increase in nutrition knowledge, with a rise in mean score from 84% preintervention to 97% postintervention. Nurses also self-reported improved personal comfort around addressing nutritional issues with patients	

		RNs and APPs completed a second educational offering one week before implementation about correctly identifying oral nutritional supplement samples based on the patient's nutritional needs, which was provided by an industry RDN.	4.1. Instruction on how to perform the behaviour<br>9.1. Credible source	No evidence	during focused patient assessments.	
		Three RNs and two APPs completed a 10-question preintervention knowledge quiz before the education session. Four RNs and three APPs completed the same quiz again six weeks after project implementation.	2.7. Feedback on outcome(s) of behaviour	None		

Poveda 2018 (27)	1. Intentions<br>2. Environmental context and resources	Team leader provided training, developed the tools to be used as strategies to increase compliance, collect the data, and supervise the implementation program.	2.1. Monitoring of behaviour by others without feedback	None	**Adoption:** Adherence to one criterion (''a multidisciplinary meeting to plan for patient discharge has been held early after admission'') was low both before and after the implementation program - partly due to pre-existing clinical commitments that did not allow new activities; baseline compliance: 0 - 22% post implementation: 67% against audit criterion<br><br>**Acceptability, Fidelity:** MDT members did not accept the need for a discussion specifically addressing the problems relating to the use of home nasoenteric feeding, therefore, for the purposes of this study, this MDT discussion focused only on specific medical problems encountered during hospitalization.	Aligns with existing mapping
		Multidisciplinary meetings explaining the goals of the implementation project and the required authorization.	6.3. Information about others approval	None		

Wang 2014 (62)	1. Environmental context and resources<br>2. Skills<br>3. Knowledge	The project team organized education sessions for all nurses in the ward about malnutrition and preventive measures. Nurses were allowed to educate the patients after they had passed the test following this education session, which was developed by the audit team.	4.1. Instruction on how to perform the behaviour<br>5.1. Information about health consequences	LINKS (Kn:4.1)<br>LINKS (Kn:5.1)<br>LINKS (Sk:4.1)	**Adoption, Fidelity:** improvements in all criteria of the audit; (pre vs. post)<br>Criteria 1: validated screening tool 0% vs 100%;<br>Criteria 2: Screen at admission 0% vs 100%;<br>Criteria 3: Patients' education 63% vs 100%;<br>Criteria 4: Dietary counselling 0% vs 100%;<br>Criteria 5: Support plan 0% vs 90%;<br><br>**Effectiveness, Timeliness:** All gastrointestinal (GI) cancer patients were assessed immediately using the NRS-2002	Aligns with existing mapping

		Develop educational material for GI cancer patients and their caregivers. It was provided to each GI cancer patient in the first day of a course of chemotherapy. A BMI check tool was included and patients were asked to record their weight.	2.4. Self-monitoring of outcome(s) of behaviour<br>4.1. Instruction on how to perform the behaviour<br>5.1. Information about health consequences<br>9.1. Credible source	LINKS (Kn:4.1)<br>LINKS (Kn:5.1)<br>LINKS (Sk:4.1)	Chinese edition on admission; All GI cancer patients received educational material; Improved use of Hospital Information System and decreased time for nurses to inform medical staff<br><br>**Effectiveness:** The results of the re-test showed a distinct improvement in nutrition knowledge, and all ward nurses learned to apply NRS-2002 to detect nutrition problems on admission of patients, and provide a brief patient education.	
		Improvements with the Hospital Information System for doctors and nurses.	7.1. Prompts/cues	LINKS (ECR:7.1)		

Zeng 2023 (63)	N/A	The PDCA cycle is a multi-step approach to nutrition management that identifies problems, defines the process, analyses the causes, and selects improvement options, which constantly reinforces the importance of nutrition management to patients and medical staffs.	1.2. Problem solving	N/A	**Safety:** The incidence of nutritional risk, the grade of malnutrition, and the grade of oral mucositis were less in the PDCA group than those in the control group ($p < 0.05$).	N/A
		The team members checked the completion of the nutrition plan, diet records, nutritional status, and occurrence of oral mucositis every week.	2.1. Monitoring of behaviour by others without feedback<br>2.5. Monitoring of outcome(s) of behaviour without feedback	N/A		
Zhang 2020 (30)	1. Environmental	Establishing MDT with dietician	12.2. Restructuring the social environment	LINKS (ECR:12.2)	**Adoption, Fidelity:** Increased compliance with best practice across all measured criteria	Aligns with

	context and resources<br>2. Knowledge	Created training/education programs for staff	4.1. Instruction on how to perform the behaviour	LINKS (Kn:4.1)	Baseline compliance rates: (criteria 1 0%, 2 0%, 3 76%, 4 82%, 5 76%) improved to at least 82% for all 5 criteria as a result of the intervention<br><br>**Effectiveness:** Completion of baseline nutritional assessment improved from 0% compliance at baseline to 94% at follow-up audit; Involvement of MDT in care of patients with or at risk of oral mucositis increased from 0 to 86%; Patients receiving recommended pain management increased from 76% to 94%; Patients (or their caregivers) receiving education regarding nutritional intake increased from 82% to 100%; Patients receiving adequate nutritional intake increased from 76% to 96%.	existing mapping
		Nurse-led provision of nutritional education to patients	4.1. Instruction on how to perform the behaviour	LINKS (Kn:4.1)		

Zhang 2021 (28)	1. Environmental context and resources<br>2. Knowledge<br>3. Intentions	Conduct training on the prevention and management of underfeeding in EN for nurses, and carry out a knowledge test, only those who scored 90 or more will be deemed qualified.	4.1. Instruction on how to perform the behaviour	LINKS (Kn:4.1)	**Adoption, Fidelity:** Compliance for all audit criteria increased post implementation:<br>Criteria 1, 2,<br>and 7 improved from 0 to 100%, criteria 3 and 4 increased from 80 to 100%, criterion 5 improved from 20<br>to 90%, criterion 6 increased from 0 to 80%, and criterion<br>8 improved from 80 to 100%.<br>(Table 1 includes criteria details)<br><br>**Effectiveness:** The feeding rate of enteral nutrition postimplementation was higher than the baseline audit on the third day, 54.29%(±12.01) vs. 42.89%(±10.63);<br><br>**Timeliness**: The postoperative fasting time of patients at postimplementation was shortened, and patients began enteral nutrition at the average of 56.00 (±11.51) h after the surgery, which was	Aligns with existing mapping
		Develop a standardised functional exercise protocol, and transmit the video, picture and text to patients through social media on the mobile phone, after which they were guided and trained on site by the responsible nurses.	4.1. Instruction on how to perform the behaviour	LINKS (Kn:4.1)		

		Compile the health education manual of EN and transmit it to patients via WeChat, followed by face-to-face meeting with nurse to introduce the benefits (health & financial), encourage patients to express their discomfort	5.1. Information about health consequences<br>5.3. Information about social and environmental consequences	LINKS (Kn:5.1)<br>LINKS (In:5.1)<br>LINKS (Kn:5.3)	significantly earlier than that at the baseline audit 75.20 (±15.09, P<0.05).<br><br>**Safety:** the feeding intolerance postimplementation (26.67%) was lower than the baseline audit (76.67%) within 1 week of enteral nutrition. incidence of underfeeding<br>was lower (30%, n = 30) than the baseline audit (76.67%, n = 30).	
