# Supplementary material for: Identifying Behaviour Change Techniques in Cancer Nutrition Interventions and Their Implementation Contexts: A Systematic Review
Source: Nutrients. 2026 Jan 12;18(2):242. doi: 10.3390/nu18020242 (PMC12845379; doi:10.3390/nu18020242)
Supplement: Supplementary file 1 [file nutrients-18-00242-s001.zip › Supplementary file S4.pdf]

Supplementary file S4. Behaviour Change Techniques identified in implementation strategies that aligned with positive patient outcomes.

BCT	Satisfaction	Symptomatology	Function	Mortality
1.1. Goal setting (behaviour)	1	2	2	1
1.3. Goal setting (outcome)	1	1	2	1
1.4. Action planning	2	1	1	1
1.5. Review behaviour goal(s)	1	2	2	1
1.6. Discrepancy between current behaviour and goal	1	1	1	1
1.7. Review outcome goal(s)	1	3	2	1
2.2. Feedback on behaviour	2	2	2	1
2.3. Self-monitoring of behaviour	1	3	2	1
2.5. Monitoring of outcome(s) of behaviour without feedback	-	-	1	-
2.7. Feedback on outcome(s) of behaviour	3	4	2	1
4.1. Instruction on how to perform the behaviour	5	5	2	1
5.1. Information about health consequences	3	5	2	1
7.1. Prompts/cues	1	1	-	-
9.1. Credible source	3	3	1	-
11.1. Pharmacological support	-	-	1	-
12.1. Restructuring the physical environment	1	-	-	-
12.2. Restructuring the social environment	1	1	-	-
12.5. Adding objects to the environment	1	2	2	1
